# Local ablation vs partial nephrectomy in T1N0M0 renal cell carcinoma: An inverse probability of treatment weighting analysis

**DOI:** 10.1002/cam4.3433

**Published:** 2020-09-05

**Authors:** Lei Shi, Yan He, Chang Liu, Xiaoyuan Qian, Zhixian Wang

**Affiliations:** ^1^ Department of Urology The First Affiliated Hospital of Zhengzhou University Zhengzhou University Zhengzhou China; ^2^ Department of Urology The First Affiliated Hospital of Xinxiang Medical University Xinxiang Medical University Xinxiang China; ^3^ Department of Urology Tongji Medical College Tongji Hospital Huazhong University of Science and Technology Wuhan China

**Keywords:** ablation, outcomes, partial nephrectomy, renal cell carcinoma, SEER, surgery

## Abstract

**Objective:**

To compare the survival outcomes of local ablation (LA) and partial nephrectomy (PN) for T1N0M0 renal cell carcinoma (RCC).

**Method:**

We identified 38,155 T1N0M0 RCC patients treated with PN or LA in 2004‐2016 from the retrospective Surveillance, Epidemiology, and End Results databases. Among them, there were 4656 LA and 33,499 PN. A Cox proportional hazards regression model, cause‐specific Cox regression and Fine and Gray sub‐distribution hazard ratio (sHR) with inverse probability of treatment weighting (IPTW) adjusting was utilized to compare the effects of LA vs PN on all‐, RCC‐, and non‐RCC–caused mortality.

**Results:**

Within the IPTW analysis, patients who underwent PN experienced a better overall survival (OS) (HR, 1.56; 95% CI, 1.40‐1.74; *P* < .001) and cancer‐specific survival (CSS) (HR, 2.21; 95% CI, 1.62‐2.98; *P* < .001) than LA patients. In the subgroup of patients >85 years (HR, 1.14; 95% CI, 0.73‐1.79, *P* = .577), chromophobe RCC (HR, 1.68; 95% CI, 0.94‐3.00, *P* = .078), and tumor size <2 cm (HR, 1.21; 95% CI, 0.95‐1.53, *P* = .126), the OS showed no significant difference between LA and PN. No significant difference in CSS between LA and PN was observed in the subgroup of chromophobe RCC (HR, 0.34; 95% CI, 0.03‐3.97, *P* = .389), and tumor size <2 cm (HR, 1.83; 95% CI, 0.92‐3.64, *P* = .084). For patients >85 years (sHR, 0.89; 95% CI, 0.52‐1.27, *P* = .520) and tumor size <2 cm (sHR, 1.14; 95% CI, 0.94‐1.38, *P* = .200), the non‐RCC–specific mortality was not significantly different in PN and LA cohorts, however, for the chromophobe RCC, the LA showed a worse non‐RCC mortality than PN (HR, 1.72; 95% CI, 1.06‐2.79, *P* = .028).

**Conclusion:**

PN showed a better prognosis than LA in T1N0M0 RCC treatment, but LA and PN showed a comparable OS in elderly patients (>85), small RCC (<2 cm) and chromophobe RCC.

## Graphical abstract

To compare the prognosis of local ablation (LA) and partial nephrectomy (PN) for T1N0M0 renal cell carcinoma (RCC) in the era of active surveillance and minimally invasive PN. Overall, PN showed a better OS and CSS than LA in T1N0M0 RCC treatment, but for elder age (>85 years), small tumors (<2 cm), and chromophobe RCC, both approaches showed no significant difference.

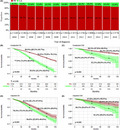

## 1 INTRODUCTION

The incidence rates of renal cell carcinoma (RCC) have been increasing in recent decades, especially with more detection of incidental small RCC due to ubiquitous abdominal imaging in current clinical practice.^1^ In the past, radical nephrectomy (RN) by open or laparoscopic approach was the standard traditional treatment for RCC. With an increased understanding of the natural history and biology of RCC, guidelines have recently shifted toward the adoption of partial nephrectomy (PN) for the treatment of T1a and expanding selected T1b RCC when the technique is feasible^2^ because PN can provide an equivalent oncological control or superior kidney functional outcome.^3^, ^4^, ^5^, ^6^, ^7^ With the rapid development of laparoscopic and robot‐assisted surgical techniques, PN is mainly minimally invasive, which further promotes its application.

However, given the complications^8^, ^9^ induced by surgical intervention and potential overtreatment for localized small kidney masses,^10^ more effort should be made to minimize the risk of intraoperative complications during PN, and alternative approaches are necessary for high‐risk patients. Local ablation (LA), mainly radiofrequency ablation and cryoablation,^2^ has gradually gained acceptance as an option for localized small RCC. It tends to be used in patients who are elderly, have a severe cardiopulmonary disease, one kidney, hereditary RCC, or those who are poor candidates for PN or RN.^11^, ^12^, ^13^ Although some studies with small sample sizes reported that LA was an effective and safe alternative treatment for T1a ^14^, ^15^ and even suitable for T1b RCC,^16^, ^17^ there is a lack of sufficient evidence and guidelines to support the use of LA as a standard treatment, so treatment selection remains an empirical process.

The primary purpose of this study was to compare the survival prognoses following PN and LA for local T1N0M0 RCC. Given individual differences in demographical and clinical characteristics and the heterogeneity of biological characteristics among RCCs, we also conducted subgroup analyses and prognostic risk assessment for patients with local T1N0M0 RCC and compared the survival outcomes of LA and PN in different prognostic risk groups. The goal of this is to better screen people who are ideal candidates for LA with survival equivalent to or better than PN and to expand the indications of LA to benefit more people.

## 2 PATIENTS AND METHODS

### 2.1 Database and patient identification

In our retrospective study, all cohorts were obtained from the Surveillance, Epidemiology, and End Results (SEER) cancer database sponsored by the United States National Cancer Institute covering cancer patients’ demographical and clinical characteristics, cancer incidence, treatment, and survival outcomes from different cancer registries. The SEER 18 registries were used for patient selection, representing ~28% of the US population, and the patients' characteristics are comparable to the general population (https://seer.cancer.gov/). All case lists were identified and selected using SEER *Stat software (version 8.3.6). Since SEER data are anonymized, the need for institutional review board approval was waived.

**FIGURE 1:**
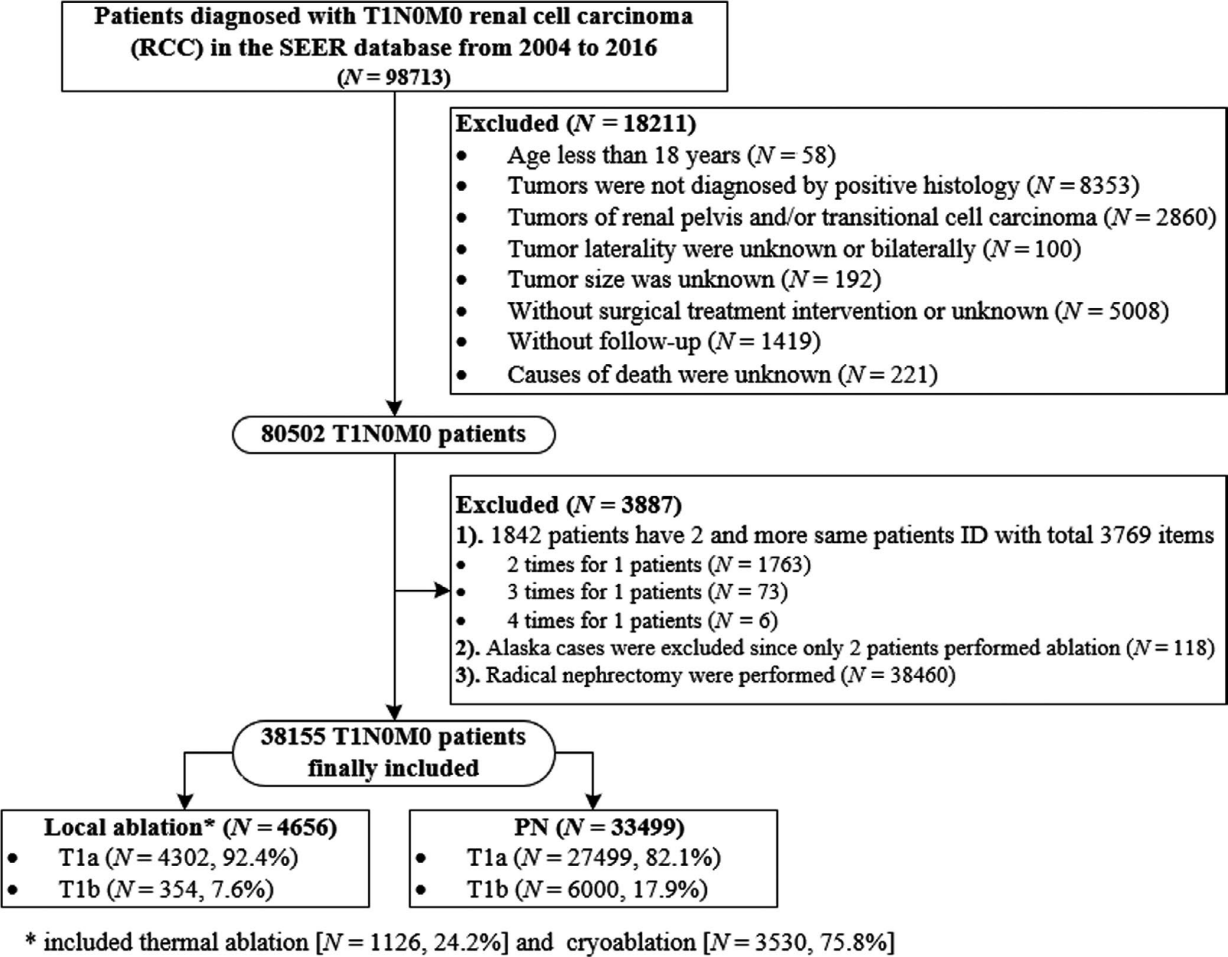
Flow chart for the data screening

Figure 1 presents a flowchart of data selection from the SEER database. All histologically confirmed T1N0M0 RCC (ICD‐O‐3 code C64.9) patients who underwent LA (RX Summ‐‐Surg Prim Site code in SEER database: 13, 15, and 23) or PN (RX Summ‐‐Surg Prim Site code in SEER database: 30) between 2004 and 2016 were included in the present study. Patients were excluded from the analysis for the following reasons: (a) age <18 years old; (b) no histological diagnosis or only by the autopsy/death certification; (c) tumor laterality was unknown or bilateral; (d) histology of tumor suspected origin from the renal pelvis, such as translational cell type; (e) lacking detailed information on tumor size, follow‐up, cause of death, or patients who underwent RN. For individual patient IDs with multiple records, the first was included. Derived American Joint Committee on Cancer 6th (2004‐2009), 7th (2010‐2015), and SEER Combined Stage (2016+) were used for RCC tumor node metastasis (TNM) staging classification in our study.

### 2.2 Study covariables

We collected several demographical and clinical variables: the year of diagnosis, family income quartile, population, region, marital status, population density, age at diagnosis, sex, race/ethnicity (White, Black, and Others [American Indian/Alaska Native, Asian Native, and Asian/Pacific Islander]), and history of malignancy prior to primary RCC diagnosis. The tumor‐related characteristics included tumor size (cm) and histological cell type for RCC (clear cell, papillary, chromophobe, and other undefined cell types), tumor grade (well‐differentiated [grade 1], moderately differentiated [grade 2], poorly differentiated [grade 3], and undifferentiated [grade 4]), and tumor laterality.

### 2.3 Outcomes for analysis

The primary outcome of interest was overall survival (OS) and cancer‐specific survival (CSS, in the study, is RCC‐specific survival). The cause of death was determined following the cases list from the SEER database. Patients who died from non‐RCC causes were identified as competing for events for mortality by RCC. The survival interval was defined as the time from the date of RCC diagnosis to the date of death (events occurred) or last contact (censor).

### 2.4 Statistical analysis

Continuous variables and categorical variables are described as the mean (SD) and frequencies (%), respectively. The Student's *t*‐test or Wilcoxon rank‐sum test was used to compare continuous variables between groups, depending on whether the continuous variable data were normally distributed or not, respectively. The categorical variables were compared by the chi‐square test or Fisher's exact test. The reverse Kaplan‐Meier method was used to calculate the median follow‐up time.

In the nonrandomized studies, the effect of treatment on outcomes can be impacted by treatment‐selection bias in which the treated cohort systematically differs from the control cohort. To account for section bias and cofounding factors between the LA vs PN groups when comparing outcomes, weighted propensity score (PS) analysis was performed to balance differences in baseline demographical and clinical variables between patients who received LA and PN. First, a PS for each individual was calculated as the predicted probability of intervention with LA compared to PN from a multivariable logistic regression that included baseline confounding factors associated with survival outcomes. Then, we included all baseline characteristics for weighted PS analysis. The inverse probability of treatment weighting (IPTW) approach was used to generate the propensity model.^18^ The weights are based on each individual's probability of receiving LA given the confounders, which is known as the PS, the weights are 1/PS for the LA participants and 1/(1 − PS) for the PN participants. The IPTW method is based on comparing the distribution of measured baseline covariates between treated and control groups in the sample weighted by the estimated inverse probability of treatment.^19^ In brief, the IPTW method uses the principle of the standardization method to assign a corresponding weight to each research object through the PS value, so that the PS distributions are comparable between groups. This approach is a standardized method based on individuals that reduce the influence of confounding factors. Comparing the difference between the groups in the IPTW sample was made using standardized mean differences (SMDs), with a threshold <0.1 indicating a nonclinically meaningful difference.

The Kaplan‐Meier method using log‐rank statistics was used to compare OS and CSS between the LA and PN groups for the unweighted and IPTW populations. An IPTW Cox proportional hazard regression model and cause‐specific regression analysis were used to evaluate the risk factors of overall and cause‐specific mortality with and without adjusting confounders. Finally, we performed subgroup analyses for the impact of different treatments (PN vs LA) in each subgroup population for the overall and cancer‐specific mortality of patients with T1N0M0 RCC. In the subgroup analysis, we deleted incomplete data. Each subgroup was adjusted by IPTW to ensure that there was no statistical difference in basic data between the PN and LA groups. We also applied the sensitivity analysis for subgroup data without deleting the incomplete information.

All analyses were conducted using the R statistical package (v.3.6.3; R Foundation for Statistical Computing; https://www.r‐project.org). All *P* values are two sided, and *P* < .05 indicates statistical significance.

## 3 RESULTS

### 3.1 Patient baseline characteristics and treatments

**FIGURE 2:**
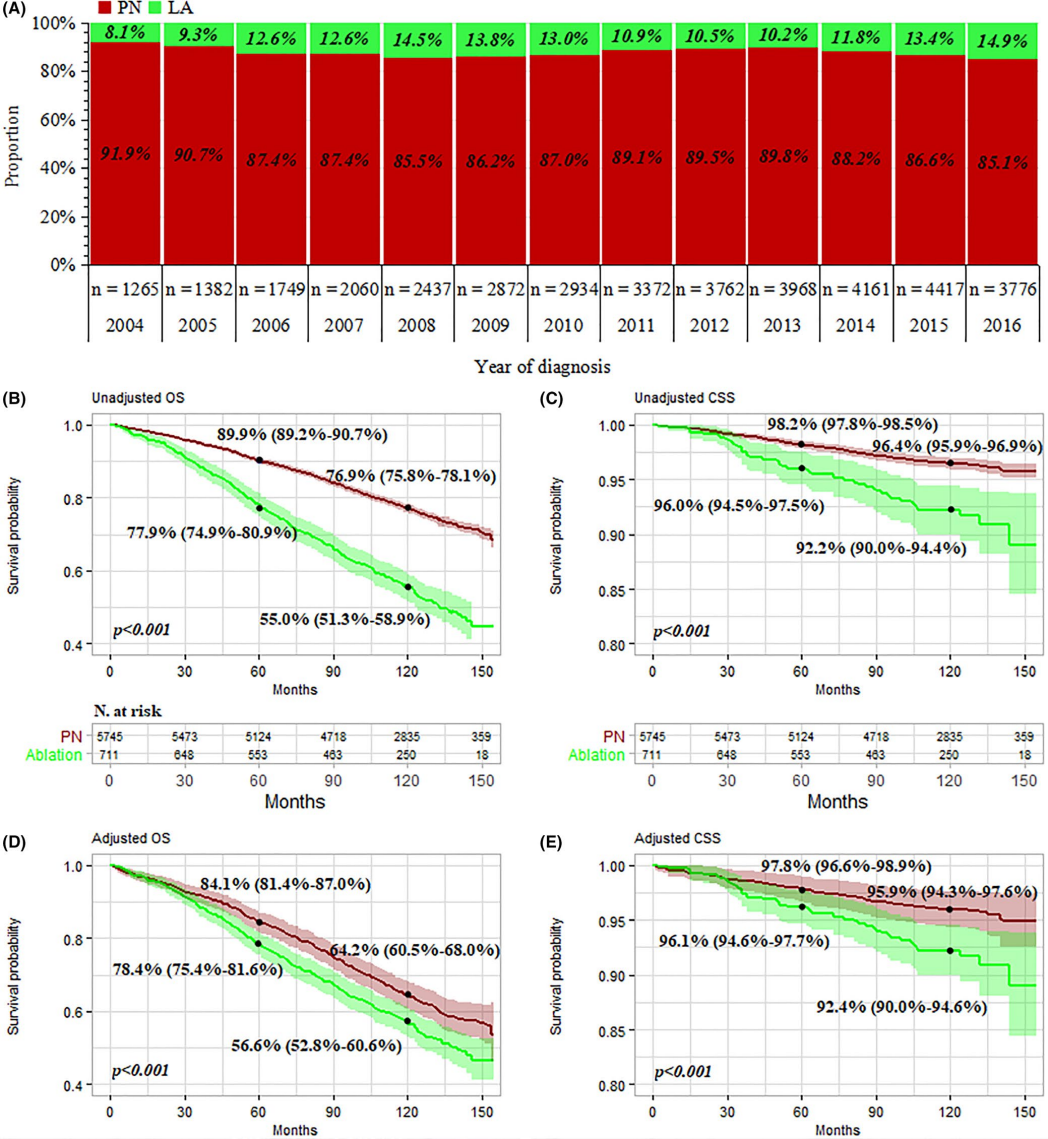
Surgery distribution, overall survival (OS) and cancer‐specific survival (CSS) of patients with T1N0M0 renal cell carcinoma underwent partial nephrectomy (PN) vs local ablation (LA). A, Proportion of surgery with the year at diagnosis; (B,C) unadjusted OS, and CSS, respectively; D,E, adjusted OS and CSS, respectively (renal cell carcinoma diagnoses between 2004 and 2007 with at least 10 y follow‐up were analyzed using the Kaplan‐Meier method)

Of the 98,713 T1N0M0 RCC patients in the databases between 2004 and 2016, 38,155 met the selection criteria; 4656 and 33,499 underwent LA and PN, respectively (Figure 1). The proportion of LA increased with the year of diagnosis, from 8.1% in 2004 to 14.9% in 2016 (Figure 2A). Table 1 lists patients’ demographical and clinicopathologic characteristics before and after propensity adjustment. Before IPTW adjustment, compared to the cohort of PN, patients who underwent LA were older (mean age 67.6 vs 58.8 years, *P* < .001), more 75 + years old (30.6% vs. 9.3%; *P* < .001), had a history of at least one prior cancer diagnosis (30.8% vs 18.6%; *P* < .001), were male (64.0% vs 61.9%; *P* < .006), had smaller tumor size (mean tumor size, 2.71 vs 2.95; *P* < .001), had tumor size >4 cm (7.6% vs 17.9%; *P* < .001), had less RCC with nuclear grade III+IV (5.8% vs 21.3%; *P* < .001), and more histology of RCC were other type/unknown (24.7% vs 16.6%; *P* < .001). Population density, adjusted median family incomes, and tumor laterality were not significantly different between groups (*P* = .390 for population density, *P* = .379 for adjusted family incomes, and *P* = .474 for laterality, respectively). After IPTW adjustment, there was no significant difference between groups, with SMDs < 10% for all covariables, indicating an excellent balance of baseline demographical and clinicopathologic characteristics between the PN and LA groups.

**TABLE 1:** Baseline characteristics of patients (n = 38 155) who received PN or LA

Covariables	Overall (N = 38 155)	Unweighted population				Inverse probability of imputed treatment weighted population^a^			
		PN group (N = 33 499)	LA group^b^ (N = 4656)	*P*‐value	SMD	PN group	LA group^a^	*P*‐value	SMD
Median follow‐up time, mo	55	54	55	*—*	*—*	55	56	*—*	*—*
Year at diagnosis				0.004	0.053			0.373	0.025
2004‐2007	6456 (16.9%)	5745 (17.1%)	711 (15.3%)			16.3%	15.5%		
2008‐2012	15 377 (40.3%)	13 487 (40.3%)	1890 (40.6%)			40.6%	40.5%		
2013‐2016	16 322 (42.8%)	14 267 (42.6%)	2055 (44.1%)			43.0%	44.0%		
Region				<0.001	0.235			0.994	0.005
East	16 001 (41.9%)	14 376 (42.9%)	1625 (34.9%)			36.0%	35.9%		
Northern plains	3874 (10.2%)	3131 (9.3%)	743 (16.0%)			15.5%	15.4%		
Pacific coast	16 926 (44.4%)	14 850 (44.3%)	2076 (44.6%)			44.0%	44.2%		
Southwest	1354 (3.5%)	1142 (3.4%)	212 (4.6%)			4.4%	4.5%		
Adjusted median family income				0.379	0.014			0.636	0.009
$(~74 400]	17 999 (47.2%)	15 774 (47.1%)	2225 (47.8%)			51.4%	51.8%		
$(74 400~)	20 156 (52.8%)	17 725 (52.9%)	2431 (52.2%)			48.6%	48.2%		
Insurance				<0.001	0.146			0.750	0.019
Any medicaid	3137 (8.2%)	2678 (8.0%)	459 (9.9%)			9.6%	9.8%		
Insured	29 418 (77.1%)	25 760 (76.9%)	3658 (78.6%)			78.1%	78.3%		
Uninsured	719 (1.9%)	686 (2.0%)	33 (0.7%)			0.8%	0.8%		
Unknown	4881 (12.8%)	4375 (13.1%)	506 (10.9%)			11.6%	11.1%		
Population density				0.390	0.014	13.9%	13.8%	0.860	0.003
Counties	32 857 (86.1%)	28 828 (86.1%)	4029 (86.5%)						
Urban/rural	5298 (13.9%)	4671 (13.9%)	627 (13.5%)						
Prior cancer diagnosis				<0.001	0.296			0.882	0.009
No	30 487 (79.9%)	27 265 (81.4%)	3222 (69.2%)			70.9%	71.3%		
1 only	6458 (16.9%)	5323 (15.9%)	1135 (24.4%)			23.5%	23.2%		
2 or more	1210 (3.2%)	911 (2.7%)	299 (6.4%)			5.6%	5.6%		
Marital status				<0.001	0.181			0.976	0.008
Married	24 161 (63.3%)	21 359 (63.8%)	2802 (60.2%)			60.9%	60.9%		
Never married	5719 (15.0%)	5123 (15.3%)	596 (12.8%)			13.3%	13.2%		
Separated/widowed/divorced	6346 (16.6%)	5291 (15.8%)	1055 (22.7%)			21.6%	21.5%		
Unknown	1929 (5.1%)	1726 (5.2%)	203 (4.4%)			4.2%	4.4%		
Age at diagnosis, y				<0.001	0.74			0.986	<0.001
Mean (SD)	59.8 (12.5)	58.8 (12.3)	67.6 (11.6)			66.4 (10.9)	66.4 (11.5)		
Median (IQR)	61.0 (52.0‐69.0)	60.0 (51.0‐68.0)	69.0 (60.0‐76.0)			67.0 (59.0‐75.0)	67.0 (59.0‐75.0)		
Age at diagnosis, y				<0.001	0.704			0.984	0.007
≦59	17 627 (46.2%)	16 570 (49.5%)	1057 (22.7%)			25.2%	25.4%		
60‐74	15 986 (41.9%)	13 811 (41.2%)	2175 (46.7%)			48.5%	48.4%		
75‐84	4101 (10.7%)	2913 (8.7%)	1188 (25.5%)			22.9%	22.7%		
85+	441 (1.2%)	205 (0.6%)	236 (5.1%)			3.1%	3.1%		
Race				<0.001	0.087			0.998	0.0043
White	31 242 (81.9%)	27 372 (81.7%)	3870 (83.1%)			82.7%	82.8%		
Black	4350 (11.4%)	3797 (11.3%)	553 (11.9%)			12.1%	12.0%		
Other	2214 (5.8%)	2005 (6.0%)	209 (4.5%)			4.7%	4.7%		
Unknown	349 (0.9%)	325 (1.0%)	24 (0.5%)			0.5%	0.5%		
Sex				0.006	0.044			0.949	0.001
Female	14 431 (37.8%)	12 756 (38.1%)	1675 (36.0%)			63.2%	63.3%		
Male	23 724 (62.2%)	20 743 (61.9%)	2981 (64.0%)			36.8%	36.7%		
Grade				<0.001	0.824			0.959	0.005
I + II	26 568 (69.6%)	23 859 (71.2%)	2709 (58.2%)			65.7%	65.5%		
III + IV	7419 (19.4%)	7149 (21.3%)	270 (5.8%)			6.6%	6.6%		
Unknown	4168 (10.9%)	2491 (7.4%)	1677 (36.0%)			27.7%	27.9%		
Laterality				0.474	0.011	51.3%	51.2%	0.909	0.002
Left	18 468 (48.4%)	16 191 (48.3%)	2277 (48.9%)						
Right	19 687 (51.6%)	17 308 (51.7%)	2379 (51.1%)						
Histological type				<0.001	0.277			0.958	0.015
ccRCC	22 441 (58.8%)	19 936 (59.5%)	2505 (53.8%)			55.8%	56.3%		
paRCC	6520 (17.1%)	5719 (17.1%)	801 (17.2%)			17.6%	17.4%		
chRCC	2473 (6.5%)	2275 (6.8%)	198 (4.3%)			4.9%	4.7%		
Other RCC	1389 (3.6%)	1277 (3.8%)	112 (2.4%)			2.7%	2.6%		
Undefined	5332 (14.0%)	4292 (12.8%)	1040 (22.3%)			19.1%	19.0%		
Tumor size, cm				<0.001	0.206			0.389	0.015
Mean (SD)	2.92 (1.26)	2.95 (1.29)	2.71 (0.943)			2.7 (1.10)	2.7 (0.96)		
Median (IQR)	2.70 (2.00‐3.60)	2.70 (2.00‐3.70)	2.60 (2.00‐3.20)			2.5 (2.00‐3.30)	2.6 (2.00‐3.25)		
Tumor size group, cm				<0.001	0.332			0.878	0.015
≦2	11 067 (29.0%)	9757 (29.1%)	1310 (28.1%)			29.3%	28.9%		
2‐3	12 829 (33.6%)	10 902 (32.5%)	1927 (41.4%)			40.7%	40.5%		
3‐4	7905 (20.7%)	6840 (20.4%)	1065 (22.9%)			21.9%	22.4%		
4‐7	6354 (16.7%)	6000 (17.9%)	354 (7.6%)			8.1%	8.3%		

Note

All the range of follow‐up time was 1‐155 mo. Wilcoxon rank‐sum test for the variables of age and tumor size; Chi‐square test for categorical variables.

Abbreviations: ccRCC, clear cell renal cell carcinoma; chRCC, chromophobe renal cell carcinoma; CI, confidence intervals; IQR, interquartile range; LA, Local ablation; paRCC, papillary renal cell carcinoma; PN, partial nephrectomy; SD, standardized difference; SMD, standardized mean difference.

^a^ Propensity scores were estimated in multivariable logistic regression models with all covariables, and inverse probability of imputed treatment weighted data was then created.

^b^ Including 1126 thermal ablation and 3530 cryoablation.

### 3.2 Follow‐up and survival outcomes

The median follow‐up was 54 months in the PN group vs 55 months in the LA group, the range was 1‐155 months. A total of 961 (20.6%) patients in the LA group and 3182 (9.4%) in the PN group died, and 155 (3.3%) in the LA group were RCC‐specific mortality compared with 474 (1.4%) in the PN group. To accurately estimate survival, RCC diagnoses between 2004 and 2007 with at least 10 years follow‐up were further analyzed using the Kaplan‐Meier method (Figure 2B‐E, the survival curve of 2004‐2016 was presented in the Figure S1). Before propensity adjustment, within the entire cohort, the median OS was significantly higher with PN compared with LA (NA vs 134 months; *P* < .001, Figure 2B). The OS rates at 5 and 10 years were 89.9% and 76.9% with PN and 77.9% and 55.0% with LA, respectively (Figure 2B). After IPTW adjustment, PN was still associated with improved OS. The OS rates at 5 and 10 years were 84.1% and 64.2% with PN vs 78.4% and 56.6% with LA, respectively (Figure 2D). The 5‐ and 10‐year CSS rates were >90.0% for both groups with and without IPTW adjustment, but the PN group fared better than the LA group (*P* < .001, Figure 2C,E).

Within different subgroups (before IPTW‐adjusted data presented in Table S1 and after IPTW‐adjusted data presented in Table S2). The 5‐ and 10‐year CSS rates were excellent with >80%, and most >90% for patients undergoing either PN or LA with or without IPTW adjustment. However, the OS was impacted by higher age, larger tumor size, and history of prior cancer, which were associated with 10‐year OS < 40%. For OS estimated by the Kaplan‐Meier method, all subgroups had significant differences in CSS for PN and LA (*P* < .001), except subgroups of patients with any Medicaid (*P* = .079), age >85 years (*P* = .420), and histology of chromophobe renal carcinoma (*P* = .240).

### 3.3 Treatment as a predictor for survival outcomes

Prognostic factors associated with OS and CSS in the overall cohort before IPTW adjustment are listed in Table S3. Compared with PN, LA was significantly associated with shorter OS and CSS (adjusted HR for OS, 1.66; 95% CI, 1.3‐1.81; *P* < .001; adjusted HR for CSS, 2.41; 95% CI, 1.95‐2.97; *P* < .001). After IPTW adjustment, LA had 1.56‐ and 2.21‐fold risk of all‐cause mortality and RCC‐caused mortality, respectively, in comparison with PN after adjusting all other variables (adjusted HR for all‐cause mortality, 1.56; 95% CI, 1.40‐1.74; *P* < .001; adjusted HR for CSS, 2.21; 95% CI, 1.63‐2.98; *P* < .001) (Table S4). Patients with lower family income, no insurance, a history of cancer, unmarried/separated/widowed/divorced, elder age, male gender, higher tumor grade, histology of papillary RCC, and increased tumor size had significantly increased risk of all‐cause mortality. However, insurance, marital status, and sex were not independent predictors for RCC‐caused mortality.

**FIGURE 3:**
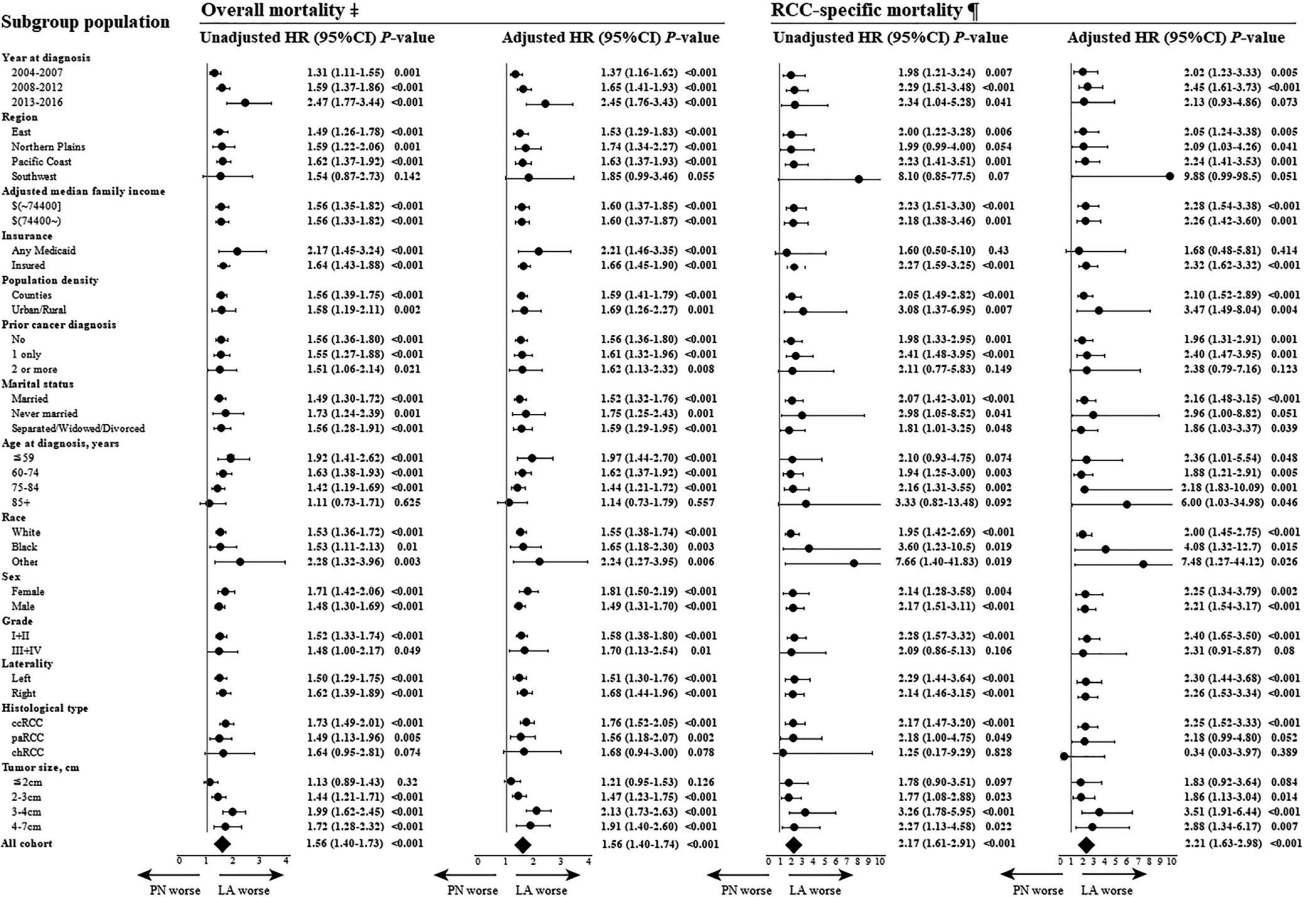
Subgroup analysis for the risk of overall and cancer‐specific mortality between different treatments of partial nephrectomy (PN) (as a Ref. [1]) vs local ablation (LA) in patients with T1N0M0 renal cell carcinoma (inverse probability of imputed treatment weighted population‐based on surgery for each subgroup). ccRCC, clear cell renal cell carcinoma; chRCC, chromophobe renal cell carcinoma; CI, confidence interval; HR, hazard ratio; paRCC, papillary renal cell carcinoma; sHR, sub‐distribution hazard ratio. ^‡^Univariable (unadjusted model) and multivariate (full covariables adjusted model) Cox regression analysis in each subgroup cohort. ^¶^Univariable (unadjusted model) and multivariate (full covariables adjusted model) cause‐specific Cox regression analysis in each subgroup cohort

The effect of PN was consistent across subgroups before IPTW (Table S5). However, after IPTW adjustment, within subgroups of patients older than 85, histology of chromophobe RCC, and tumor size <2 cm, patients who underwent LA showed no significant difference for overall mortality compared with PN treatment, besides, there was no significant difference in CSS between LA and PN treatment in the subgroup of chromophobe RCC (HR adjusted, 0.34; 95% CI, 0.03‐3.97, *P* = .389), and tumor size <2 cm (HR adjusted, 1.83; 95% CI, 0.92‐3.64, *P* = .084) (Figure 3). Table 2 presents the results of non‐RCC–specific death for LA compared with PN in the subgroup of age, tumor size and histological types. For patients >85 years (sHR, 0.89; 95% CI, 0.52‐1.27, *P* = .520) and tumor size <2 cm (sHR, 1.14; 95% CI, 0.94‐1.38, *P* = .200), the non‐RCC–specific mortality was not significantly different between the PN and LA cohorts. And for the chromophobe RCC, the LA still showed a worse non‐RCC mortality risk than PN (HR adjusted, 1.72; 95% CI, 1.06‐2.79, *P* = .028). Similar results were obtained with the IPTW‐adjusted cause‐specific Cox regression model.

**TABLE 2:** Subgroup analysis for the impact of different treatments of partial nephrectomy (as a Ref. [1]) vs local ablation in subgroup population on non‐RCC–specific mortality (a competing death) of patients with T1N0M0 renal cell carcinoma

	Model 1^a^		Model 2^b^	
	Adjusted sHR (95% CI)	*P*‐value	Adjusted HR (95% CI)	*P*‐value
All cohort	1.51 (1.38‐1.65)	<0.001	1.56 (1.43‐1.71)	<0.001
Age at diagnosis, y				
≦59	2.14 (1.70‐2.68)	<0.001	1.92 (1.50‐2.46)	<0.001
60‐74	1.62 (1.42‐1.84)	<0.001	1.58 (1.37‐1.82)	<0.001
75‐84	1.37 (1.17‐1.59)	<0.001	1.36 (1.15‐1.60)	<0.001
85+	0.89 (0.62‐1.27)	0.520	0.96 (0.64‐1.43)	0.837
Tumor size, cm				
≦2	1.14 (0.94‐1.38)	0.200	1.12 (0.91‐1.37)	0.279
2‐3	1.44 (1.24‐1.67)	<0.001	1.41 (1.21‐1.64)	<0.001
3‐4	1.92 (1.61‐2.28)	<0.001	1.97 (1.64‐2.36)	<0.001
4‐7	1.82 (1.41‐2.33)	<0.001	1.73 (1.31‐2.28)	<0.001
Histological type				
ccRCC	1.72 (1.52‐1.94)	<0.001	1.66 (1.47‐1.89)	<0.001
paRCC	1.42 (1.15‐1.77)	0.002	1.0.48 (1.16‐1.88)	0.001
chRCC	1.72 (1.06‐2.79)	0.028	1.77 (1.06‐2.97)	0.030

Abbreviations: ccRCC, clear cell renal cell carcinoma; chRCC, chromophobe renal cell carcinoma; CI, confidence interval; HR, Hazard ratio; paRCC, papillary renal cell carcinoma; sHR, Sub‐distribution hazard ratio.

^a^ Results were computed by Fine and Grey regression using the data of crude unweighted population for each subgroup.

^b^ Results were computed by cause‐special Cox regression using the data of inverse probability of imputed treatment weighted population for each subgroup.

## 4 DISCUSSION

PN has become the most common surgery for localized RCC.^20^, ^21^ With advances in laparoscopic and robotic‐assisted technologies, most operations can be performed in a minimally invasive manner. Laparoscopic or robot‐assisted PN has been widely recognized and recommended by the surgical community.^22^ Although laparoscopic or robot‐assisted PN offers a good trade‐off between minimally invasive and organ‐sparing procedures to achieve a win‐win effect, surgery still needs to be performed under general anesthesia. This may be associated with serious complications in patients with more comorbidities and poor physical tolerance. A prior study showed that about 5% of patients undergoing PN for a clinically localized renal tumor developed an intraoperative complication,^8^ and higher American Anesthesiologists Score, complex RCC (eg, endophytic RCC), and the surgical technique were independent predictors of trifecta outcomes.^9^ To reduce complications caused by PN and improve the quality of life for RCC patients, LA has gradually gained acceptance as an alternative to PN for patients with localized small RCC.^2^ Our research validates the trend that the application of LA has increased over time, from 8.1% in 2004 to 14.9% in 2016. Previous studies compared the early or long‐term oncological outcomes, renal function, and complications between patients treated with LA and PN^12^, ^14^, ^16^, ^17^, ^23^, ^24^, ^25^, ^26^, ^27^, ^28^, ^29^, ^30^, ^31^, ^32^, ^33^, ^34^, ^35^, ^36^, ^37^, ^38^, ^39^, ^40^; some are summarized in Table 3. These studies suggested that when selecting patients, those treated with LA or PN showed comparable oncological outcomes and equivalent or better kidney function preservation.

**TABLE 3:** Overview of current literature on local ablation and PN outcomes

Study	Years of pooled pts.	Treatment	No. of pts	Mean/median age (y)	Size (cm)	Follow‐up (mo)	Conclusions
^a^, ^b^Yu et al^24^	2006‐2017	MWA LPN	185 1770	63.2 50.9	2.3 2.3	40.6	1. No significant differences regarding oncologic outcomes or complications between percutaneous microwave ablation and laparoscopic PN for patients with cT1a renal cell carcinoma. 2. Percutaneous microwave ablation led to smaller renal function changes and reduced blood loss.
^b^Shapiro et al^25^	2000‐2018	MWA PN RN	40 74 211	69 58 59	4.4 4.7 5.0	34 35 49	No difference in 5‐y metastasis‐free survival or cancer‐specific survival was found among microwave ablation, partial nephrectomy, or radical nephrectomy.
^a^, ^b^Rembeyo et al^26^	2010‐2016	RPN CRA RFA	36 55 11	60 72 84	4.5 4.6 4.2	23.7 19.9 51.3	1. No significant difference in renal preservation between the groups. 2. The adjusted hazard ratio for local recurrence‐free survival was significantly shorter for the cryoablation group.
^b^Zhou et al^27^	2006‐2016	RFA CRA MWA	244 26 27	73 68 69	2.4 2.4 2.2	24	Radiofrequency ablation, cryoablation, and microwave ablation are equivalent at 2 y for the treatment of T1a renal cell carcinoma for the therapeutic outcome, renal function stability, and low adverse event rate.
^a^, ^b^Pecoraro et al^29^	2004‐2015	CRA PN	242 5521	71 61	4.69 5.1	38	A 2.5‐fold increase in cancer‐specific mortality when cryoablation was performed in patients with T1b renal cell carcinoma.
^b^Park et al^30^	2005‐2014	RFA PN	62 53	58 53	2.14 2.75	60 68	The groups did not differ in terms of eGFR change 1‐2 wk after surgery or at the last follow‐up or 5‐y survival rates.
^a^, ^b^Kitley et al^31^	1998‐2012	CRA PN	6701 51 135	68 58	2.5 2.4	NA	Cryotherapy had lower overall survival than PN for tumors > 2 cm.
^a^, ^b^Andrews et al^32^	2000‐2011	CRA RFA PN	187 180 1055	72 72 62	2.8 1.9 2.4	75.6 90 112.8	1. For cT1a patients, clinically relevant differences between PN and ablation are unlikely, and treatment choice should involve shared decision making. 2. For cT1b patients, death from renal cell carcinoma was more common with cryoablation, and large differences in this outcome cannot be ruled out.
^c^Alam et al^33^	2009‐2018	PN RN AT AS	231 41 27 339	61.3 69.3 71.8 70.6	2.4 3.1 2.1 1.8	36	1. PN and ablation are preferred over radical nephrectomy when intervention is indicated for small renal mass. 2. Active surveillance is a reasonable option for select patients, given comparable oncologic and mental health outcomes.
^b^Zhou et al^34^	2006‐2016	RFA CRA MWA	305 41 38	72 72 69	2.7 2.9 2.5	NA	CT‐guided percutaneous microwave ablation is comparable to radiofrequency ablation or cryoablation for the treatment of stage T1N0M0 renal cell carcinoma (concerning treatment response) and is associated with shorter treatment times and less sedation than radiofrequency ablation or cryoablation.
^b^Xing et al^35^	2002‐2011	PN RN TA AS	2820 4522 898 1978	71.9 73.9 75.9 NA	NA	NA	For T1aN0M0 renal cell carcinoma, thermal ablation confers cancer‐specific and overall survival rates similar to those seen with surgical management, with significantly fewer adverse outcomes at 1 y after the procedure and similar rates of secondary cancer events compared with surgery.
^b^Talenfeld et al^12^	2006‐2011	AT PN RN	456 1748 2106	NA	NA	44 51 55	1. Percutaneous ablation may result in oncologic outcomes similar to those of RN, but with less long‐term renal insufficiency and markedly fewer periprocedural complications. 2. Compared with partial nephrectomy, percutaneous ablation may be associated with slightly shorter disease‐specific survival but fewer periprocedural complications.
^a^, ^b^Park et al^37^	2008‐2016	RPN RFA	63 63	NA	NA	24.6 21	Partial nephrectomy provides a higher recurrence‐free survival rate than radiofrequency ablation.
^a^, ^b^Caputo et al^38^	1999‐2014	CRA PN	31 161	68 61	4.3 5.0	30.1 13.0	Patients treated with cryoablation for cT1b renal tumors had a significantly higher rate of local cancer recurrence at 1 y compared to those treated with partial nephrectomy.
^b^Pantelidou et al^39^	2005‐2013	RFA RPN	63 63	61 54	2.11 2.88	47.5 18.5	Radiofrequency ablation demonstrated fewer perioperative complications and better preservation of renal function, whereas PN had an insignificantly lower local recurrence rate.
^b^Larcher et al^40^	2000‐2009	AT PN	561 2289	76 72	2.8 2.6	35	Ablative therapies offer a short‐term protective effect from acute kidney injury. The short‐term rates of any dialysis treatment are similar after either ablative therapies or partial nephrectomy. At the long‐term assessment, renal function detriment rates are not different between ablative therapies and partial nephrectomy.
^b^Thompson et al^16^	2000‐2011	PN RFA CRA	1057 180 187	62 72 72	2.4 1.9 2.8	60 35 16.8	Recurrence‐free survival was similar for PN and percutaneous ablation patients. Metastasis‐free survival was superior for PN and cryoablation patients when compared with radiofrequency ablation for cT1a patients. Overall survival was superior after PN.
^b^Chang et al^17^	2006‐2010	RFA PN	27 29	64.0 56.9	4.7 5.2	65.9 70.2	Radiofrequency ablation is an effective treatment option that provides 5‐y overall survival, disease‐specific survival, and disease‐free survival rates comparable to that of PN, as well as better renal function preservation than PN for T1b renal cell carcinoma.
^b^Olweny et al^14^	1998‐2005	RFA PN	37 37	63.8 54.8	2.1 2.5	78 73.2	Radiofrequency ablation yields comparable long‐term oncologic outcomes to nephron‐sparing surgery.

Abbreviations: AS, active surveillance; AT, ablative therapies; CRA, cryoablation; CT, computed tomography; eGFR, estimated glomerular filtration rate; MWA, microwave ablation, laparoscopic partial nephrectomy; PA, percutaneous ablation; PN, partial nephrectomy; RFA, radiofrequency ablation; RPN, robot‐assisted partial nephrectomy; TA, thermal ablation.

^a^ Propensity score matching study.

^b^ Retrospective study.

^c^ Prospective cohort study.

The primary aim of our study was to compare the prognoses of LA and PN after controlling for clinical baseline characteristics using IPTW adjustment. It was found that the patients undergoing PN still have a longer OS or CSS, but for patients >85 years, RCC <2 cm, and histology of chromophobe RCC, the LA cohort did not have significant differences for OS and CSS compared with the PN cohort. Patient selection is crucial for treatment decision making, and a prior study concluded that patients at high risk of complications (elder age, higher CCI, acute/chronic kidney injury, larger tumor size) may benefit the most from LA.^41^ Also, both general and treatment‐specific complications can occur following LA, the increased risk of complications was attributed to patient‐related (increased age and higher CCI) and tumor‐related (increased size and next to renal sinus) factors, and included both urological and nonurological etiologies.^2^ The incidence of complications was reported to be lower or comparable to that of PN^11^, ^35^, ^38^, ^42^; nevertheless, some studies have found no statistical differences between PN and LA surgical complications and postoperative kidney function changes.^13^, ^38^ Major complications of ablation occurred in 3.1%‐7.4% of cases, and overall complication rates were about 14%, but adverse effect reporting has not been standardized and is prone to bias in predominantly retrospective series.^2^ Among all complications, bleeding is the most common, and cryoablation procedures showed a higher bleeding rate compared with radiofrequency ablation (4.9% vs 1.2%).^2^

Older patients often have conditions affecting other organs and systems, especially cardiopulmonary diseases, which greatly increase the risk of noncancer death. For elderly patients with short life expectancy, considering that PN or RN surgery may be poorly tolerated and carry an increased risk of postoperative complications and competing mortalities, patients and physicians tend to choose LA. The current literature on the comparison of PN and LA surgical methods are usually concentrated on the elderly patient population with a median age of ~60 years.^11^, ^16^, ^35^ We found that the prognosis of young patients receiving PN was better than that of LA patients. Besides, the patients included in previous studies were mainly elderly patients >65, with limited age stratification for older groups. We performed further stratification analysis on patients >60 and found that patients aged 60‐85 can still benefit more from PN than LA concerning OS and CSS, but there is no difference in OS for patients over 85. Age alone does not fully reflect the patient's physical health; other comorbidities and the CCI need to be considered. This is an important risk factor that affects the prognosis and was emphasized in prior studies. LA is recommended for RCC patients with CCI > 2.^41^, ^42^

Tumor size is an important sign of solid tumor TNM staging and determining surgery complexity. Our study found that only for RCC < 2 cm, there were no significant differences in OS and CSS between LA and PN surgery. LA is recommended by the American Urological Association^43^ and the European Association of Urology^5^ guidelines in patients with T1a RCC without lymph node invasion and distant metastasis. At present, there is still much controversy about LA usage for larger‐diameter tumors, such as T1bN0M0 patients undergoing LA.^16^, ^17^, ^29^, ^38^ In a high‐quality, comparative study concerning the efficacy of local cryoablation vs PN for T1b RCC treatment conducted by Caputo et al^38^ renal cryoablation had a higher rate of local cancer recurrence; however, there was no significant difference in RCC‐specific mortality or overall mortality between the cryoablation and PN groups. One of the limitations of that study is the relatively small sample size, with a total of 31 patients undergoing LA, but at a single‐center, 31 complete T1b RCC patients undergoing LA surgery provide valuable data. Besides, they observed differences in the outcomes of interest between the two surgical methods, which met certain research objectives.

A recently published study by Pecoraro suggests that in T1b RCC, LA can increase the risk of RCC‐specific death of patients by about twofold compared to PN.^29^ Studies have suggested that the application of new treatment techniques cannot take the "extreme" path, and indications should be determined using strong, evidence‐based medical data. In clinical practice, patients with T1bRCC should undergo PN as first‐line treatment whenever possible. PN operation for complex RCC can benefit from the advancement and application of robot technology. Many centers around the world can treat RCC with minimally invasive PN, which can obtain good results with regard to complications and patient quality of life. RCC size also affects the success rate of LA treatment. It has been reported that 26.5% of patients need to undergo two or more LA operations to achieve successful treatment, especially for large RCC; for tumors >4.5 cm, approximately 7.4% of patients undergo LA treatment.^44^ Mauri et al^45^ reported that among 149 patients treated with thermal ablations with a median follow‐up of 54 months, 18.1% received multiple successful ablations due to incomplete ablation, local tumor progression, distant tumor progression, or multiple tumor foci. Simultaneous use of ultrasound and computed tomography in ablation surgery can improve the ability to immediately detect RCC tissue that has not been sufficiently ablated, thereby guiding immediate secondary ablation. One of the disadvantages of image‐guided ablation is that incomplete ablation may occur, especially if the RCC is large or centrally located.^45^ It should be noted that these results are mostly from large medical institutions. In these studies, patient enrollment, technical equipment, and surgeon experience may offer certain advantages, so it is necessary to interpret these conclusions carefully.

We found no statistical difference in oncologic control between the two surgical methods in the chromophobe RCC population. One group concluded that the prognosis of clear cell RCC after LA was worse than that of nonclear cell carcinoma, which is consistent with previous research.^46^ In their study, 229 patients were included (181 clear cell RCC and 48 papillary RCC). After LA, the 5‐year disease‐free survival rates were 89.7% for clear cell RCC and 100% for papillary RCC, but there was no significant difference with respect to OS (88.4% vs 89.6%, *P* = .764). However, in LA group, the histology of clear cell RCC showed no significant difference in CSS and OS compared with nonclear cell RCC (papillary RCC vs clear cell RCC: HR_OS_, 0.88, 95% CI 0.73‐1.07, HR_CSS_, 0.91, 95% CI 0.57‐1.47; chromophobe RCC vs clear cell RCC: HR_OS_, 0.74, 95% CI 0.52‐1.06, HR_CSS_, 0.26, 95% CI 0.06‐1.07, data not shown). These results indicate that although the pathological type may not have a statistically significant effect on the prognosis of patients receiving LA, the prognosis of nonclear cell RCC is better, albeit not significantly.

A comparative study of laparoscopic LA and PN and a meta‐analysis provided fair evidence that oncologic outcomes are substantially worse for laparoscopic LA than for laparoscopic/robot‐assisted laparoscopic PN, but laparoscopic LA may be associated with improved perioperative outcomes.^23^ However, with the widespread application of laparoscopic technology and increased surgeon experience, complications can be controlled and decreased, and some anatomically complex and large volume RCC can be treated with PN in a short time, and kidney functions damage can be minimalized through techniques such as blocking and nonblocking of renal artery branches. RCC enucleation can also reduce excessive normal healthy renal parenchyma loss. Because both laparoscopic LA and PN require general anesthesia, the benefits of LA in the era of mature laparoscopic technology still need further study. However, image‐guided percutaneous LA can indeed avoid the harm caused by general anesthesia. It is a good approach for some patients who are not suitable to undergo laparoscopic PN.

In the era of active surveillance and minimally invasive PN, evidence supporting the effectiveness and safety of ablation techniques continues to be refined.^35^, ^42^, ^45^ There are still some difficulties in the promotion of LA; for example, ablation technology and equipment are not widely available in less developed areas. However, standard laparoscopic equipment and PN technology are increasingly common, and complications are decreased compared with the development period of the PN technique. Another issue is that an image follow‐up plan after ablation has not been established.

Our research also has certain limitations. The most important consideration is that our research data are retrospective, which has an inherent bias. However, to control the selection bias as much as possible and conduct case‐control studies, we used the PS weighting method to correct the baseline data in both groups. In our study, we could not adjust the CCI since the SEER database did not report it, but it is fundamental to correctly schedule the patients for a specific treatment; therefore, we used other competing mortality as a surrogate of CCI and to weight the competing risk analysis. We found that for subgroups of patients >85 years and tumor size <2 cm, non‐RCC–specific death was not significantly different, which suggested that the risks of competing events for the PN and LA cohorts were comparable in these two subgroups. However, we must interpret our results carefully, because the total cohort data suggested the LA group had a higher risk of non‐RCC–specific death compared with the PN group. This also indicated, to a certain extent, that our data still have potential biases between the two groups, even after IPTW adjustment of baseline characteristics. Since the records of surgical complications were incomplete, the article does not discuss the complications, but our main purpose was not to compare complications between methods. Rather, we focused on the comparison of OS and CSS. With the advancement of PN and LA technologies, the incidence of perioperative complications is now much lower, and the research pays more attention to long‐term patient prognosis, which is conducive to risk assessment and postoperative health consultation.

In conclusion, both PN and LA are valuable treatments for local small RCC treatment. Considering that the prognosis is generally worse following LA, we need to be cautious selecting patients to maximize the benefit from treatment. Our results indicate that age, tumor size, and histological type are important determinants for PN and LA surgical decision making.

## CONFLICT OF INTEREST

The authors declare to have no competing interest.

## AUTHORS CONTRIBUTIONS

Lei Shi and Zhixian Wang conceived the research and wrote the manuscript. Zhixian Wang registry the data. Lei Shi, Yan He, and Zhixian Wang analysis the data and prepare the figures and tables. All the authors were involved in approval of the final version.

## Supporting information

Figure S1

Table S1‐S5

## Data Availability

The raw data of this study are derived from the SEER database (https://seer.cancer.gov/), which is a publicly available database. All detailed data included in the study are available upon request by contact with the corresponding author (Zhixian Wang, SEER Username: 10062‐Nov2018).
