# Supplementary material for: Local ablation vs partial nephrectomy in T1N0M0 renal cell carcinoma: An inverse probability of treatment weighting analysis
Source: Cancer Med. 2020 Sep 5;9(21):7988–8003. doi: 10.1002/cam4.3433 (PMC7643644; doi:10.1002/cam4.3433)
Supplement: Supplementary file 1 — Figure S1 [file CAM4-9-7988-s001.docx]

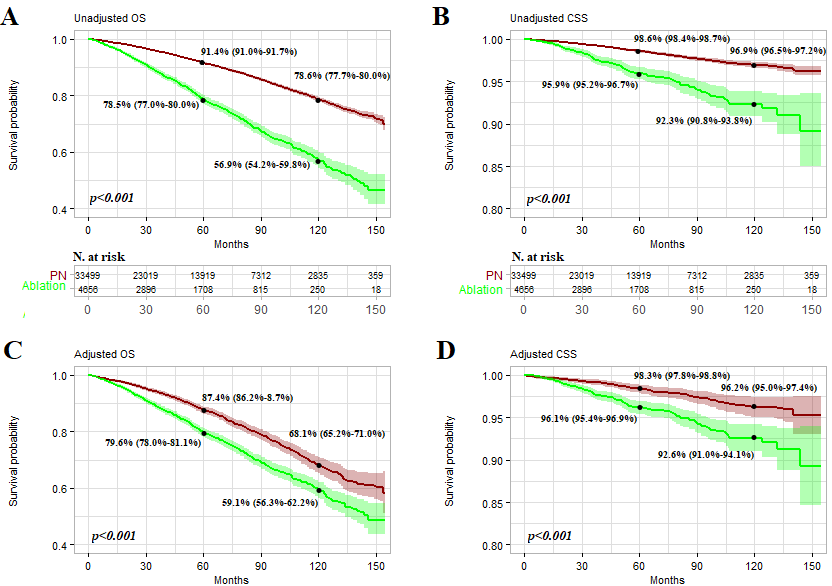


**Supplementary Figure 1.** Overall survival (OS) and cancer-specific survival (CSS) of patients with T1N0M0 renal cell carcinoma underwent partial nephrectomy (PN) vs. local ablation. A-B, unadjusted OS, and CSS, respectively; C-D, adjusted OS and CSS, respectively
