# Supplementary material for: Local ablation vs partial nephrectomy in T1N0M0 renal cell carcinoma: An inverse probability of treatment weighting analysis
Source: Cancer Med. 2020 Sep 5;9(21):7988–8003. doi: 10.1002/cam4.3433 (PMC7643644; doi:10.1002/cam4.3433)
Supplement: Supplementary file 2 — Table S1‐S5 [file CAM4-9-7988-s002.docx]

| **Supplementary Table 1**. 5-, 10-year crude overall and cancer-specific survival between two surgical methods (partial nephrectomy vs. local ablation) in each unweighted subgroup population | | | | | | | | | | | | | | | | | |
| --- | --- | --- | --- | --- | --- | --- | --- | --- | --- | --- | --- | --- | --- | --- | --- | --- | --- |
| **Subgroup population** | **Overall Survival** | | | | | | | |  | **Cancer-specific survival** | | | | | | | |
|  | **5-year** | | |  | **10-year** | | | **P**  **-value** |  | **5-year** | | |  | **10-year** | | | **P**  **-value** |
|  | **Partial nephrectomy** | **Local ablation** | **D** |  | **Partial nephrectomy** | **Local ablation** | **D** |  |  | **Partial nephrectomy** | **Local ablation** | **D** |  | **Partial nephrectomy** | **Local ablation** | **D** |  |
| **AJCC T stage** |  |  |  |  |  |  |  |  |  |  |  |  |  |  |  |  |  |
| T1a | 92.3 (91.9-92.7) | 80.0 (78.5-81.5) | 12.3% |  | 80.2 (79.3-81.1) | 58.8 (55.9-61.8) | 21.4% | <0.001 |  | 99.0 (98.9-99.2) | 96.5 (95.8-97.2) | 2.5% |  | 97.5 (97.2-97.9) | 93.0 (91.6-94.5) | 4.5% | <0.001 |
| T1b | 86.6 (85.5-87.8) | 60.7 (54.5-67.6) | 25.9% |  | 70.0 (67.6-72.5) | 34.2 (25.2-46.2) | 35.8% | <0.001 |  | 96.3 (95.7-97.0) | 88.8 (84.5-93.4) | 7.5% |  | 93.2 (91.9-94.4) | 82.4 (73.7-92.1) | 10.8% | <0.001 |
| **Region** |  |  |  |  |  |  |  |  |  |  |  |  |  |  |  |  |  |
| East | 91.1 (90.5-91.7) | 79.0 (76.5-81.5) | 12.1% |  | 78.4 (77.1-79.7) | 57.7 (53.4-62.3) | 20.7% | <0.001 |  | 98.7 (98.5-98.9) | 96.2 (95.1-97.4) | 2.5% |  | 96.9 (96.4-97.4) | 93.7 (91.8-95.7) | 3.2% | <0.001 |
| Northern Plains | 90.7 (89.5-92.0) | 76.5 (72.8-80.3) | 14.2% |  | 76.6 (73.8-79.4) | 54.6 (48.0-62.1) | 22.0% | <0.001 |  | 98.4 (97.8-98.9) | 95.8 (94.0-97.7) | 2.6% |  | 97.2 (96.2-98.2) | 91.7 (87.6-95.9) | 5.5% | <0.001 |
| Pacific Coast | 91.8 (91.2-92.3) | 78.6 (76.4-80.9) | 13.2% |  | 79.2 (77.9-80.5) | 57.0 (52.7-61.7) | 22.2% | <0.001 |  | 98.5 (98.2-98.7) | 96.0 (94.9-97.1) | 2.5% |  | 96.7 (96.1-97.2) | 92.0 (89.6-94.3) | 4.7% | <0.001 |
| Southwest | 91.9 (89.9-93.9) | 81.1 (74.4-88.4) | 10.8% |  | 79.6 (75.2-84.2) | 56.4 (42.7-74.5) | 23.2% | <0.001 |  | 98.4 (97.5-99.4) | 93.3 (88.4-98.4) | 5.1% |  | 97.7 (96.5-98.9) | 85.0 (73.6-98.1) | 12.7% | <0.001 |
| **Adjusted median family income** |  |  |  |  |  |  |  |  |  |  |  |  |  |  |  |  |  |
| $(~74400] | 90.4 (89.8-91.0) | 76.4 (74.2-78.7) | 14.0% |  | 76.2 (74.9-77.5) | 54.4 (50.4-58.7) | 21.8% | <0.001 |  | 98.4 (98.1-98.6) | 94.9 (93.8-96.1) | 3.5% |  | 96.9 (96.4-97.4) | 90.8 (88.3-93.3) | 6.1% | <0.001 |
| $(74400~) | 92.2 (91.7-92.7) | 80.3 (78.4-82.4) | 11.9% |  | 80.5 (79.4-81.6) | 59.0 (55.2-63.0) | 21.5% | <0.001 |  | 98.7 (98.5-98.9) | 96.8 (95.9-97.7) | 1.9% |  | 96.9 (96.4-97.4) | 93.6 (91.8-95.3) | 3.3% | <0.001 |
| **Insurance** |  |  |  |  |  |  |  |  |  |  |  |  |  |  |  |  |  |
| Any Medicaid¶ | 88.5 (86.8-90.3) | 72.8 (67.3-78.8) | 15.7% |  | 73.4 (68.2-79.0) | 47.6 (36.5-62.0) | 25.8% | <0.001 |  | 97.9 (97.2-98.7) | 96.3 (94.0-98.7) | 1.6% |  | 97.0 (95.8-98.2) | 94.1 (89.4-99.1) | 2.9% | 0.079 |
| Insured¶ | 92.0 (91.6-92.4) | 79.7 (78.0-81.4) | 12.3% |  | 81.2 (79.9-82.5) | 59.4 (55.3-63.8) | 21.8% | <0.001 |  | 98.7 (98.5-98.9) | 96.0 (95.2-96.9) | 2.7% |  | 96.7 (96.1-97.3) | 91.6 (89.3-93.9) | 5.1% | <0.001 |
| Uninsured | 90.1 (89.2-90.9) | 76.3 (72.6-80.1) | 13.8% |  | 77.0 (75.7-78.3) | 57.5 (53.3-62.2) | 19.5% | <0.001 |  | 98.3 (97.9-98.7) | 95.4 (93.4-97.3) | 2.9% |  | 96.8 (96.2-97.3) | 92.7 (90.1-95.3) | 4.1% | <0.001 |
| **Population density** |  |  |  |  |  |  |  |  |  |  |  |  |  |  |  |  |  |
| Counties | 91.5 (91.1-91.9) | 78.8 (77.2-80.4) | 12.7% |  | 79.0 (78.1-79.9) | 56.4 (53.4-59.6) | 22.6% | <0.001 |  | 98.6 (98.4-98.7) | 96.1 (95.4-96.9) | 2.5% |  | 96.9 (96.5-97.3) | 92.4 (90.8-94.0) | 4.5% | <0.001 |
| Urban/Rural | 90.4 (89.4-91.4) | 76.7 (72.6-81.0) | 13.7% |  | 75.8 (73.4-78.2) | 60.0 (53.4-67.4) | 15.8% | <0.001 |  | 98.6 (98.1-99.0) | 94.6 (92.3-97.0) | 4.0% |  | 96.5 (95.4-97.6) | 91.8 (87.8-95.9) | 4.7% | <0.001 |
| **Prior cancer diagnosis** |  |  |  |  |  |  |  | <0.001 |  |  |  |  |  |  |  |  | <0.001 |
| No | 93.0 (92.6-93.4) | 82.6 (81.0-84.3) | 10.4% |  | 81.7 (80.8-82.6) | 61.2 (57.8-64.7) | 20.5% | <0.001 |  | 98.9 (98.7-99.0) | 96.8 (96.1-97.6) | 2.1% |  | 97.5 (97.1-97.8) | 94.5 (93.0-96.0) | 3.0% | <0.001 |
| 1 only | 86.0 (84.9-87.1) | 72.4 (69.2-75.7) | 13.6% |  | 68.1 (65.8-70.5) | 51.7 (46.6-57.3) | 16.4% | <0.001 |  | 97.3 (96.8-97.9) | 94.4 (92.7-96.2) | 2.9% |  | 93.9 (92.8-95.2) | 87.2 (83.4-91.2) | 6.7% | <0.001 |
| 2 or more | 75.4 (72.0-79.0) | 59.6 (53.1-66.9) | 15.8% |  | 53.2 (47.6-59.6) | 34.4 (25.0-47.4) | 18.8% | <0.001 |  | 96.4 (94.8-98.0) | 91.6 (87.4-95.9) | 4.8% |  | 95.2 (92.8-97.6) | 88.4 (82.7-94.5) | 6.8% | <0.001 |
| **Marital status** |  |  |  |  |  |  |  |  |  |  |  |  |  |  |  |  |  |
| Married | 92.4 (92.0-92.8) | 80.9 (79.2-82.7) | 11.5% |  | 80.7 (79.8-81.7) | 62.2 (58.9-65.7) | 18.5% | <0.001 |  | 98.6 (98.4-98.8) | 96.0 (95.0-96.9) | 2.6% |  | 96.9 (96.5-97.3) | 92.5 (90.7-94.4) | 4.4% | <0.001 |
| Never married | 91.4 (90.4-92.4) | 77.5 (73.3-82.1) | 13.9% |  | 80.0 (77.8-82.2) | 57.5 (48.5-68.1) | 22.5% | <0.001 |  | 98.9 (98.5-99.2) | 97.2 (95.4-99.1) | 1.7% |  | 97.5 (96.7-98.4) | 92.3 (87.5-97.3) | 5.2% | <0.001 |
| Separated/Widowed/Divorced | 87.0 (85.9-88.1) | 73.5 (70.1-77.1) | 13.5% |  | 68.2 (65.8-70.6) | 44.7 (39.0-51.2) | 23.5% | <0.001 |  | 98.0 (97.6-98.5) | 95.8 (94.2-97.5) | 2.2% |  | 95.6 (94.5-96.7) | 92.4 (89.3-95.6) | 3.2% | 0.002 |
| **Age at diagnosis, years** |  |  |  |  |  |  |  |  |  |  |  |  |  |  |  |  |  |
| ≦59 | 95.4 (95.0-95.8) | 88.5 (86.2-90.9) | 6.9% |  | 89.4 (88.5-90.2) | 80.3 (76.5-84.2) | 9.1% | <0.001 |  | 99.1 (98.9-99.3) | 98.0 (97.1-99.1) | 1.1% |  | 98.2 (97.8-98.5) | 97.2 (95.7-98.7) | 1.0% | 0.003 |
| 60-74 | 89.8 (89.2-90.4) | 81.5 (79.4-83.6) | 8.3% |  | 73.6 (72.1-75.1) | 60.2 (56.0-64.8) | 13.4% | <0.001 |  | 98.3 (98.1-98.6) | 96.7 (95.7-97.7) | 1.6% |  | 95.8 (95.1-96.5) | 92.0 (89.6-94.4) | 3.8% | <0.001 |
| 75-84 | 77.3 (75.5-79.1) | 67.1 (64.1-70.2) | 10.2% |  | 45.9 (42.7-49.4) | 35.9 (31.3-41.3) | 10.0% | <0.001 |  | 96.6 (95.8-97.4) | 93.1 (91.4-94.9) | 3.5% |  | 93.5 (91.8-95.2) | 88.5 (85.3-91.9) | 5.0% | <0.001 |
| 85+ | 63.4 (55.6-72.2) | 57.5 (49.9-66.2) | 5.9% |  | 19.8 (10.5-37.4) | 17.3 (8.50-35.2) | 2.5% | 0.550 |  | 92.4 (89.7-99.0) | 88.7 (83.0-94.8) | 3.7% |  | 86.9 (78.1-96.6) | 87.1 (80.8-93.9) | -0.2% | 0.420 |
| **Race** |  |  |  |  |  |  |  |  |  |  |  |  |  |  |  |  |  |
| White | 91.3 (90.9-91.7) | 78.3 (76.7-80.0) | 13.0% |  | 78.4 (77.4-79.3) | 56.1 (53.0-59.3) | 22.3% | <0.001 |  | 98.5 (98.3-98.7) | 96.0 (95.2-96.8) | 2.5% |  | 96.7 (96.3-97.1) | 92.3 (90.7-94.0) | 4.4% | <0.001 |
| Black | 90.1 (89.0-91.3) | 78.5 (74.3-82.9) | 11.6% |  | 76.9 (74.2-79.6) | 63.8 (56.8-71.7) | 13.1% | <0.001 |  | 98.8 (98.4-99.2) | 96.4 (94.5-98.4) | 2.4% |  | 97.7 (96.9-98.6) | 94.6 (91.4-97.8) | 3.1% | <0.001 |
| Other | 94.5 (93.3-95.7) | 81.6 (75.8-87.8) | 12.9% |  | 84.7 (81.6-87.9) | 57.2 (45.3-72.3) | 27.5% | <0.001 |  | 99.2 (98.7-99.6) | 94.0 (90.2-97.9) | 5.2% |  | 97.8 (96.6-99.1) | 88.4 (82.1-95.2) | 9.4% | <0.001 |
| **Sex** |  |  |  |  |  |  |  |  |  |  |  |  |  |  |  |  |  |
| Female | 92.9 (92.3-93.4) | 80.5 (78.2-83.0) | 12.4% |  | 80.5 (79.1-81.9) | 58.8 (54.0-64.0) | 21.7% | <0.001 |  | 98.9 (98.7-99.2) | 96.6 (95.4-97.7) | 2.3% |  | 97.5 (97.0-98.0) | 93.1 (90.7-95.6) | 4.4% | <0.001 |
| Male | 90.5 (90.0-91.0) | 77.4 (75.5-79.3) | 13.1% |  | 77.4 (76.4-78.5) | 55.9 (52.5-59.4) | 21.5% | <0.001 |  | 98.3 (98.1-98.6) | 95.6 (94.6-96.5) | 2.7% |  | 96.5 (96.0-96.9) | 91.9 (90.0-93.7) | 4.6% | <0.001 |
| **Grade** |  |  |  |  |  |  |  |  |  |  |  |  |  |  |  |  |  |
| I+II | 92.1 (91.7-92.5) | 78.7 (76.8-80.7) | 13.4% |  | 79.6 (78.6-80.6) | 58.2 (54.6-62.1) | 21.4% | <0.001 |  | 99.0 (98.9-99.2) | 96.1 (95.1-97.1) | 2.9% |  | 97.5 (97.2-97.9) | 91.6 (89.4-93.8) | 5.9% | <0.001 |
| III+IV | 88.9 (88.0-89.8) | 76.5 (70.6-82.9) | 12.4% |  | 74.0 (71.9-76.2) | 56.9 (46.5-69.5) | 17.1% | <0.001 |  | 96.9 (96.4-97.4) | 92.9 (89.2-96.8) | 4.0% |  | 94.4 (93.4-95.3) | 91.6 (87.3-96.3) | 2.8% | <0.001 |
| **Laterality** |  |  |  |  |  |  |  | <0.001 |  |  |  |  |  |  |  |  | <0.001 |
| Left | 91.6 (91.1-92.2) | 79.1 (77.1-81.3) | 12.5% |  | 78.6 (77.4-79.8) | 56.9 (53.2-60.9) | 21.7% | <0.001 |  | 98.8 (98.6-99.0) | 96.5 (95.5-97.5) | 2.3% |  | 97.1 (96.6-97.6) | 92.9 (90.9-95.0) | 4.2% | <0.001 |
| Right | 91.1 (90.6-91.7) | 77.9 (75.8-80.0) | 13.2% |  | 78.6 (77.4-79.8) | 57.0 (53.1-61.3) | 21.6% | <0.001 |  | 98.4 (98.1-98.6) | 95.4 (94.3-96.5) | 3.0% |  | 96.6 (96.1-97.1) | 91.7 (89.6-93.9) | 4.9% | <0.001 |
| **Histological type** |  |  |  |  |  |  |  |  |  |  |  |  |  |  |  |  |  |
| ccRCC | 91.7 (91.2-92.2) | 77.6 (75.5-79.7) | 14.1% |  | 79.1 (78.0-80.3) | 55.9 (52.0-60.1) | 23.2% | <0.001 |  | 98.5 (98.3-98.7) | 95.4 (94.3-96.5) | 3.1% |  | 96.6 (96.2-97.1) | 91.3 (89.0-93.6) | 5.3% | <0.001 |
| paRCC | 90.0 (89.1-91.0) | 81.9 (78.5-85.5) | 8.1% |  | 77.4 (75.5-79.5) | 58.4 (51.3-66.4) | 19.0% | <0.001 |  | 98.6 (98.2-99.0) | 96.6 (95.0-98.3) | 2.0% |  | 96.5 (95.5-97.5) | 93.4 (90.5-96.5) | 3.1% | <0.001 |
| chRCC | 94.9 (93.7-96.0) | 78.4 (71.2-86.4) | 16.5% |  | 84.3 (81.4-87.3) | 64.4 (52.5-79.1) | 19.9% | <0.001 |  | 99.5 (99.1-99.9) | 98.4 (96.1-100.0) | 1.1% |  | 98.8 (98.0-99.6) | 98.4 (96.1-100.0) | 0.4% | 0.240 |
| Other RCC | 90.4 (89.5-91.3) | 78.3 (75.6-81.2) | 12.1% |  | 76.3 (74.5-78.1) | 57.2 (52.5-62.3) | 19.1% | <0.001 |  | 98.3 (97.9-98.7) | 96.1 (94.8-97.5) | 2.2% |  | 97.0 (96.3-97.7) | 92.8 (90.3-95.4) | 4.2% | <0.001 |
| **Tumor size, cm** |  |  |  |  |  |  |  |  |  |  |  |  |  |  |  |  | <0.001 |
| ≦2cm | 93.8 (93.2-94.3) | 87.0 (84.8-89.3) | 6.8% |  | 83.2 (81.9-84.6) | 71.9 (67.5-76.5) | 11.3% | <0.001 |  | 99.4 (99.2-99.6) | 97.2 (96.0-98.3) | 2.2% |  | 98.0 (97.4-98.5) | 95.7 (93.8-97.5) | 2.3% | <0.001 |
| 2-3cm | 92.1 (91.5-92.8) | 80.9 (78.8-83.2) | 11.2% |  | 80.1 (78.7-81.5) | 59.1 (54.8-63.7) | 21.0% | <0.001 |  | 98.9 (98.7-99.2) | 97.0 (96.0-98.0) | 1.9% |  | 97.5 (97.0-98.1) | 92.6 (90.3-95.0) | 4.9% | <0.001 |
| 3-4cm | 90.4 (89.5-91.3) | 69.8 (66.4-73.3) | 20.6% |  | 74.9 (72.8-77.1) | 43.6 (38.1-49.9) | 31.3% | <0.001 |  | 98.6 (98.2-98.9) | 94.7 (92.9-96.5) | 3.9% |  | 96.8 (96.0-97.6) | 90.1 (86.6-93.7) | 6.7% | <0.001 |
| PN, partial nephrectomy; LA, Local ablation; AJCC, American Joint Committee on Cancer System; D, Difference.  Survival function was estimated by the Kaplan-Meier method;Difference was computed by survival rate for PN minus survival for LA.  The numbers of subgroup cohort used for the inverse probability of imputed treatment weighted were according to Table 1, the standard mean difference between the cohort of partial nephrectomy and local ablation were less than 10% after weighting. For overall survival estimated by Kaplan–Meier method, except age more than 85 years subgroup did not significantly differ between partial nephrectomy and local ablation group (P= 0.550), all other subgroups were showed significant difference (P< 0.001); for cancer-specific survival, except subgroups of patients with any Medicaid (P= 0.079), age more than 85 years (P= 0.420), and patients with histology of chromophobe renal carcinoma (P= 0.240), all other subgroups present significantly differ between partial nephrectomy and local ablation group (P< 0.001) ǂ Propensity scores were estimated in multivariable logistic regression models with all other covariables, and inverse probability of imputed treatment weighted data were then created for each subgroup cohort, the standardized differences for all variables were less than 0.1. ¶ data just included from the year of 2007 and more. | | | | | | | | | | | | | | | | | |

| **Supplementary Table 2**. 5-, 10-year crude overall and cancer-specific survival between two surgical methods (partial nephrectomy vs. local ablation) in each weightedǂ subgroup population | | | | | | | | | | | | | | | | | | |
| --- | --- | --- | --- | --- | --- | --- | --- | --- | --- | --- | --- | --- | --- | --- | --- | --- | --- | --- |
| **Subgroup population** | **Overall Survivalǂ** | | | | | | | |  | **Cancer-specific survivalǂ** | | | | | | | | |
|  | **5-year** **(%)** | | |  | **10-year (%)** | | | **P**  **-value** |  | **5-year (%)** | | |  | **10-year (%)** | | | **P**  **-value** |  |
|  | **PN group** | **LA group** | **D** |  | **PN group** | **LA group** | **D** |  |  | **PN group** | **LA group** | **D** |  | **PN group** | **LA group** | **D** |  |  |
| **AJCC T stage** |  |  |  |  |  |  |  |  |  |  |  |  |  |  |  |  |  |  |
| T1a | 88.2 (87.0-89.6) | 81.2 (79.7-82.8) | 7.0% |  | 69.2 (66.3-72.3) | 61.3 (58.3-64.4) | 7.9% | <0.001 |  | 98.5 (98.0-99.0) | 96.7 (96.0-97.5) | 1.8% |  | 96.6 (95.5-97.8) | 93.4 (91.8-94.9) | 3.2% | <0.001 |  |
| T1b | 77.1 (71.5-83.2) | 61.4 (55.0-68.5) | 15.7% |  | 53.5 (43.8-65.2) | 35.7 (26.6-47.8) | 17.8% | <0.001 |  | 96.2 (93.6-99.0) | 89.3 (84.8-94.0) | 6.9% |  | 90.8 (84.9-97.3) | 82.6 (73.7-92.6) | 8.2% | <0.001 |  |
| **Region** |  |  |  |  |  |  |  |  |  |  |  |  |  |  |  |  |  |  |
| East | 87.2 (85.1-89.3) | 79.9 (77.4-82.5) | 7.3% |  | 68.9 (64.7-73.3) | 60.1 (55.7-64.8) | 8.8% | <0.001 |  | 98.5 (97.7-99.3) | 96.4 (95.2-97.6) | 2.1% |  | 96.5 (94.8-98.2) | 94.0 (92.0-96.0) | 2.5% | <0.001 |  |
| Northern Plains | 87.8 (84.7-91.0) | 77.9 (74.0-82.0) | 9.9% |  | 61.1 (53.2-70.0) | 56.6 (49.7-64.6) | 4.5% | <0.001 |  | 98.2 (97.0-99.5) | 95.9 (94.0-97.9) | 2.3% |  | 94.5 (90.8-98.4) | 91.3 (86.8-96.2) | 3.2% | <0.001 |  |
| Pacific Coast | 87.8 (85.9-89.8) | 79.6 (77.3-82.0) | 8.2% |  | 68.7 (64.2-73.5) | 59.2 (54.6-64.2) | 9.5% | <0.001 |  | 98.2 (97.4-99.0) | 96.2 (95.0-97.4) | 2.0% |  | 96.3 (94.5-98.3) | 92.5 (90.2-95.0) | 3.8% | <0.001 |  |
| Southwest | 87.8 (81.6-94.4) | 82.8 (75.9-90.4) | 5.0% |  | 73.0 (60.0-88.7) | 57.1 (42.9-76.1) | 15.9% | <0.001 |  | 99.3 (97.5-100.0) | 94.2 (89.3-99.4) | 5.1% |  | 99.0 (96.8-100.0) | 85.3 (73.5-99.2) | 13.7% | <0.001 |  |
| **Adjusted median family income** |  |  |  |  |  |  |  |  |  |  |  |  |  |  |  |  |  |  |
| $(~74400] | 86.1 (84.2-88.0) | 77.2 (74.9-79.6) | 8.9% |  | 65.6 (61.5-70.0) | 56.9 (52.8-61.3) | 8.7% | <0.001 |  | 97.8 (96.9-98.6) | 95.1 (93.9-96.3) | 2.7% |  | 96.1 (94.5-97.7) | 90.8 (88.2-93.6) | 5.3% | <0.001 |  |
| $(74400~) | 88.9 (87.2-90.6) | 81.8 (79.7-83.9) | 7.1% |  | 70.1 (66.2-74.2) | 60.9 (56.9-65.2) | 9.2% | <0.001 |  | 98.9 (98.3-99.5) | 97.1 (96.1-98.0) | 1.8% |  | 96.4 (94.8-98.1) | 94.0 (92.1-95.8) | 2.4% | <0.001 |  |
| **Insurance** |  |  |  |  |  |  |  |  |  |  |  |  |  |  |  |  |  |  |
| Any Medicaid¶ | 87.0 (82.2-92.2) | 72.7 (66.6-79.4) | 14.3% |  | 70.6 (59.5-83.7) | 51.6 (40.2-66.3) | 19.0% | <0.001 |  | 97.9 (96.0-99.9) | 97.1 (94.7-99.5) | 0.8% |  | 97.7 (95.5-100.0) | 95.1 (90.2-100.0) | 2.6% | 0.079 |  |
| Insured¶ | 88.2 (86.7-89.6) | 81.0 (79.3-82.8) | 7.2% |  | 72.4 (68.4-76.5) | 61.6 (57.2-66.2) | 10.8% | <0.001 |  | 98.4 (97.9-99.0) | 96.2 (95.3-97.1) | 2.2% |  | 96.1 (94.4-97.8) | 92.0 (89.6-94.4) | 4.1% | <0.001 |  |
| Uninsured | 84.1 (80.8-87.4) | 76.7 (73.0-80.6) | 7.4% |  | 63.3 (58.9-68.0) | 58.0 (53.6-62.8) | 5.3% | <0.001 |  | 98.0 (96.7-99.3) | 95.5 (93.5-97.5) | 2.5% |  | 96.3 (94.5-98.2) | 92.8 (90.2-95.4) | 3.5% | <0.001 |  |
| **Population density** |  |  |  |  |  |  |  |  |  |  |  |  |  |  |  |  |  |  |
| Counties | 87.5 (86.1-88.9) | 79.9 (78.2-81.6) | 7.6% |  | 68.0 (65.0-71.3) | 58.9 (55.8-62.2) | 9.1% | <0.001 |  | 98.3 (97.8-98.9) | 96.4 (95.6-97.2) | 1.9% |  | 96.1 (94.8-97.4) | 92.6 (90.9-94.3) | 3.5% | <0.001 |  |
| Urban/Rural | 87.3 (83.9-90.9) | 77.8 (73.6-82.3) | 9.5% |  | 68.5 (61.3-76.6) | 60.6 (53.8-68.4) | 7.9% | <0.001 |  | 98.2 (96.7-99.6) | 94.4 (91.9-96.9) | 3.8% |  | 96.9 (94.1-99.7) | 91.9 (88.0-96.0) | 5.0% | <0.001 |  |
| **Prior cancer diagnosis** |  |  |  |  |  |  |  | <0.001 |  |  |  |  |  |  |  |  | <0.001 |  |
| No | 90.2 (88.8-91.6) | 83.4 (81.7-85.1) | 6.8% |  | 71.2 (67.9-74.8) | 63.4 (59.9-67.0) | 7.8% | <0.001 |  | 98.7 (98.2-99.3) | 97.0 (96.2-97.8) | 1.7% |  | 96.7 (95.4-98.1) | 94.7 (93.1-96.3) | 2.0% | <0.001 |  |
| 1 only | 83.1 (80.2-86.1) | 73.2 (69.8-76.7) | 9.9% |  | 62.7 (57.2-68.8) | 52.9 (47.5-58.9) | 9.8% | <0.001 |  | 97.5 (96.3-98.7) | 94.4 (92.5-96.3) | 3.1% |  | 94.7 (91.9-97.6) | 86.9 (82.8-91.2) | 7.8% | <0.001 |  |
| 2 or more | 72.3 (65.2-80.2) | 61.6 (54.2-70.1) | 10.7% |  | 51.1 (39.7-65.9) | 36.4 (25.5-52.0) | 14.7% | <0.001 |  | 96.0 (92.4-99.6) | 92.7 (88.2-97.4) | 3.3% |  | 95.6 (91.4-100.0) | 89.3 (82.6-96.5) | 6.3% | 0.003 |  |
| **Marital status** |  |  |  |  |  |  |  |  |  |  |  |  |  |  |  |  |  |  |
| Married | 88.3 (86.7-89.9) | 82.3 (80.4-84.1) | 6.0% |  | 71.1 (67.7-74.7) | 64.2 (60.7-67.9) | 6.9% | <0.001 |  | 98.3 (97.6-98.9) | 96.4 (95.5-97.3) | 1.9% |  | 96.3 (94.8-97.8) | 93.1 (91.2-95.0) | 3.2% | <0.001 |  |
| Never married | 88.4 (85.0-92.1) | 78.8 (74.4-83.5) | 9.6% |  | 72.9 (65.1-81.5) | 58.8 (49.9-69.3) | 14.1% | <0.001 |  | 99.2 (98.2-100.0) | 97.4 (95.5-99.2) | 1.8% |  | 96.7 (93.0-100.0) | 92.2 (87.2-97.4) | 4.5% | <0.001 |  |
| Separated/Widowed/Divorced | 84.1 (81.1-87.2) | 73.3 (69.6-77.2) | 10.8% |  | 56.6 (50.2-63.8) | 46.9 (41.0-53.8) | 9.7% | <0.001 |  | 97.8 (96.5-99.0) | 95.4 (93.5-97.3) | 2.4% |  | 95.2 (92.6-98.0) | 92.1 (88.7-95.6) | 3.1% | 0.002 |  |
| **Age at diagnosis, years** |  |  |  |  |  |  |  |  |  |  |  |  |  |  |  |  |  |  |
| ≦59 | 94.2 (92.5-96.0) | 88.8 (86.5-91.2) | 5.4% |  | 86.8 (83.0-90.7) | 80.5 (76.7-84.4) | 6.3% | <0.001 |  | 99.3 (98.6-99.9) | 98.1 (97.1-99.1) | 1.2% |  | 98.1 (96.6-99.6) | 97.3 (95.8-98.8) | 0.8% | 0.003 |  |
| 60-74 | 88.9 (87.1-90.6) | 81.9 (79.8-84.1) | 7.0% |  | 72.5 (68.5-76.7) | 60.5 (56.1-65.3) | 12.0% | <0.001 |  | 98.3 (97.5-99.0) | 97.0 (96.0-98.0) | 1.3% |  | 96.0 (94.2-97.9) | 92.0 (89.5-94.6) | 4.0% | <0.001 |  |
| 75-84 | 79.2 (76.0-82.6) | 68.9 (65.2-72.7) | 10.3% |  | 46.2 (40.1-53.1) | 40.1 (34.4-46.6) | 6.1% | <0.001 |  | 97.1 (95.7-98.5) | 93.7 (91.7-95.8) | 3.4% |  | 94.1 (90.9-97.4) | 88.9 (85.0-93.0) | 5.2% | <0.001 |  |
| 85+ | 68.6 (59.1-79.7) | 57.7 (46.9-71.0) | 11.2% |  | 17.5 (5.4-56.5) | 24.4 (11.6-51.4) | -6.5% | 0.550 |  | 98.7 (96.4-100) | 87.4 (78.8-96.9) | 11.3% |  | 94.0 (86.0-100) | 86.8 (77.9-96.6) | 7.2% | 0.420 |  |
| **Race** |  |  |  |  |  |  |  |  |  |  |  |  |  |  |  |  |  |  |
| White | 87.1 (85.7-88.6) | 79.4 (77.7-81.1) | 7.7% |  | 67.0 (63.8-70.3) | 58.2 (55.1-61.6) | 8.8% | <0.001 |  | 98.2 (97.6-98.7) | 96.2 (95.4-97.1) | 2.0% |  | 96.0 (94.7-97.3) | 92.6 (90.9-94.3) | 3.4% | <0.001 |  |
| Black | 87.9 (84.4-91.6) | 80.3 (76.0-84.8) | 7.6% |  | 71.6 (64.4-79.6) | 65.9 (58.6-74.3) | 5.7% | <0.001 |  | 99.1 (98.0-100.0) | 96.8 (94.9-98.8) | 2.3% |  | 97.7 (95.1-100.0) | 94.8 (91.5-98.2) | 2.9% | <0.001 |  |
| Other | 90.6 (85.7-95.8) | 81.3 (75.1-88.0) | 9.3% |  | 81.4 (72.4-91.6) | 61.8 (49.6-76.9) | 19.6% | <0.001 |  | 99.1 (97.6-100.0) | 93.8 (89.7-98.0) | 5.3% |  | 98.5 (95.2-100.0) | 89.2 (82.8-96.1) | 9.3% | <0.001 |  |
| **Sex** |  |  |  |  |  |  |  |  |  |  |  |  |  |  |  |  |  |  |
| Female | 90.0 (88.1-91.9) | 80.9 (78.4-83.4) | 9.1% |  | 69.4 (64.6-74.6) | 60.5 (55.6-65.9) | 8.9% | <0.001 |  | 98.4 (97.6-99.2) | 96.6 (95.4-97.8) | 1.8% |  | 96.8 (95.2-98.5) | 92.9 (90.4-95.6) | 3.9% | <0.001 |  |
| Male | 85.9 (84.3-87.6) | 78.9 (77.0-80.9) | 7.0% |  | 67.2 (63.7-70.9) | 58.4 (54.9-62.1) | 8.8% | <0.001 |  | 98.2 (97.6-98.9) | 95.9 (94.9-96.9) | 2.3% |  | 95.9 (94.3-97.5) | 92.4 (90.5-94.3) | 3.5% | <0.001 |  |
| **Grade** |  |  |  |  |  |  |  |  |  |  |  |  |  |  |  |  |  |  |
| I+II | 87.1 (85.5-88.7) | 78.9 (77.0-80.9) | 8.2% |  | 66.3 (62.5-70.3) | 58.4 (54.7-62.3) | 7.9% | <0.001 |  | 98.5 (97.9-99.1) | 96.1 (95.1-97.1) | 2.4% |  | 96.1 (94.5-97.7) | 91.6 (89.4-93.9) | 4.5% | <0.001 |  |
| III+IV | 84.0 (78.7-89.7) | 76.5 (70.6-83.0) | 7.5% |  | 63.3 (52.7-75.9) | 56.9 (46.5-69.5) | 6.4% | <0.001 |  | 96.7 (94.1-99.5) | 92.9 (89.2-96.8) | 3.8% |  | 94.7 (90.1-99.6) | 91.6 (87.3-96.3) | 3.1% | <0.001 |  |
| **Laterality** |  |  |  |  |  |  |  | <0.001 |  |  |  |  |  |  |  |  | <0.001 |  |
| Left | 87.5 (85.7-89.3) | 80.3 (78.2-82.5) | 7.2% |  | 67.6 (63.6-71.9) | 59.4 (55.5-63.6) | 8.2% | <0.001 |  | 98.6 (98.0-99.3) | 96.6 (95.6-97.7) | 2.0% |  | 97.2 (95.8-98.7) | 93.3 (91.2-95.4) | 3.9% | <0.001 |  |
| Right | 87.4 (85.6-89.2) | 78.8 (76.7-81.1) | 8.6% |  | 68.7 (64.8-72.9) | 58.9 (54.8-63.4) | 9.8% | <0.001 |  | 98.0 (97.2-98.8) | 95.6 (94.5-96.8) | 2.4% |  | 95.3 (93.5-97.2) | 91.9 (89.7-94.2) | 3.4% | <0.001 |  |
| **Histological type** |  |  |  |  |  |  |  |  |  |  |  |  |  |  |  |  |  |  |
| ccRCC | 87.4 (85.7-89.2) | 78.1 (76.0-80.3) | 9.3% |  | 68.9 (64.8-73.3) | 57.7 (53.6-62.1) | 11.2% | <0.001 |  | 98.2 (97.5-98.9) | 95.6 (94.5-96.8) | 2.6% |  | 95.3 (93.4-97.3) | 91.6 (89.2-94.0) | 3.7% | <0.001 |  |
| paRCC | 88.9 (86.0-91.9) | 83.3 (79.9-86.9) | 5.6% |  | 69.8 (63.0-77.4) | 61.3 (54.1-69.4) | 8.5% | <0.001 |  | 98.4 (97.1-99.6) | 96.8 (95.2-98.6) | 1.6% |  | 96.7 (93.7-99.7) | 94.0 (91.0-97.0) | 2.7% | <0.001 |  |
| chRCC | 89.7 (84.2-95.5) | 78.4 (71.1-86.5) | 11.3% |  | 70.8 (59.1-84.9) | 64.9 (52.9-79.7) | 5.9% | <0.001 |  | 99.5 (98.2-100.0) | 98.3 (96.0-100.0) | 1.2% |  | 96.8 (91.9-100.0) | 98.3 (96.0-100.0) | -1.5% | 0.240 |  |
| Other RCC | 85.8 (83.1-88.6) | 80.7 (77.7-83.9) | 5.1% |  | 64.1 (58.9-69.7) | 60.6 (55.5-66.3) | 3.5% | <0.001 |  | 98.1 (97.0-99.2) | 96.5 (95.1-97.9) | 1.6% |  | 96.7 (95.0-98.4) | 93.1 (90.3-95.9) | 3.6% | <0.001 |  |
| **Tumor size, cm** |  |  |  |  |  |  |  |  |  |  |  |  |  |  |  |  |  |  |
| ≦2cm | 90.3 (88.2-92.4) | 88.5 (86.3-90.9) | 1.8% |  | 73.1 (68.1-78.5) | 73.9 (69.5-78.6) | -0.8% | <0.001 |  | 98.9 (98.2-99.6) | 97.6 (96.5-98.7) | 1.3% |  | 97.3 (95.3-99.5) | 96.0 (94.1-98.0) | 1.3% | <0.001 |  |
| 2-3cm | 87.8 (85.9-89.9) | 81.8 (79.5-84.1) | 6.0% |  | 67.8 (63.2-72.7) | 61.2 (56.7-66.1) | 6.6% | <0.001 |  | 98.4 (97.6-99.2) | 97.2 (96.2-98.3) | 1.2% |  | 95.7 (93.5-97.9) | 93.3 (90.9-95.7) | 2.4% | <0.001 |  |
| 3-4cm | 85.8 (82.9-88.7) | 71.2 (67.5-75.0) | 14.6% |  | 63.3 (57.4-69.9) | 46.3 (40.2-53.3) | 17.0% | <0.001 |  | 98.2 (97.1-99.3) | 94.7 (92.7-96.6) | 3.5% |  | 96.9 (95.0-98.8) | 89.3 (85.3-93.5) | 7.6% | <0.001 |  |
| PN, partial nephrectomy; LA, Local ablation; AJCC, American Joint Committee on Cancer System; D, Difference; CI, confidence intervals; ccRCC, clear cell renal cell carcinoma; paRCC, papillary renal cell carcinoma; chRCC, chromophobe renal cell carcinoma; CI, confidence intervals. Survival function was estimated by the Kaplan-Meier method; Difference was computed by survival rate for PN minus survival for LA.  The numbers of subgroup cohort used for the inverse probability of imputed treatment weighted were according to Table 1, the standard mean difference between the cohort of partial nephrectomy and local ablation were less than 10% after weighting. Propensity scores were estimated in multivariable logistic regression models with all other covariables, and inverse probability of imputed treatment weighted data were then created for each subgroup cohort, the standardized differences for all variables were less than 0.1.  ǂ Describe overall Survival (95%CI) and cancer-specific survival (95%CI). ¶ data just included from the year of 2007 and more. | | | | | | | | | | | | | | | | | | |

| **Supplementary Table 3**. Prognostic factors for overall and cancer-specific mortality of T1N0M0 renal cell carcinoma patients with treatment of partial nephrectomy and local ablation (unweighted population). | | | | | | | | | | | | | |
| --- | --- | --- | --- | --- | --- | --- | --- | --- | --- | --- | --- | --- | --- |
| **Covariables** | **Overall mortality ǂ** | | | | |  |  | **RCC-specific mortality ¶** | | | | | |
|  | **Unadjusted**  **HR (95%CI)** | ***P-value*** |  | **Adjusted**  **HR (95%CI)** | ***P-value*** |  |  | **Unadjusted**  **HR (95%CI)** | ***P-value*** |  | **Adjusted**  **HR (95%CI)** | ***P-value*** |  |
| **Year at diagnosis** |  |  |  |  |  |  |  |  |  |  |  |  |  |
| 2004-2007 | 1 reference |  |  | 1 reference |  |  |  | 1 reference |  |  | 1 reference |  |  |
| 2008-2012 | 0.88 (0.82-0.94) | <0.001 |  | 0.93 (0.84-1.02) | 0.125 |  |  | 0.82 (0.68-0.98) | 0.032 |  | 0.72 (0.57-0.92) | 0.007 |  |
| 2013-2016 | 0.78 (0.70-0.88) | <0.001 |  | 0.80 (0.70-0.92) | 0.001 |  |  | 0.79 (0.60-1.04) | 0.098 |  | 0.66 (0.48-0.90) | 0.010 |  |
| **Region** |  |  |  |  |  |  |  |  |  |  |  |  |  |
| East | 1 reference |  |  | 1 reference |  |  |  | 1 reference |  |  | 1 reference |  |  |
| Northern Plains | 1.15 (1.05-1.27) | 0.005 |  | 1.02 (0.92-1.13) | 0.679 |  |  | 1.15 (0.88-1.50) | 0.311 |  | 0.96 (0.74-1.26) | 0.777 |  |
| Pacific Coast | 0.96 (0.90-1.03) | 0.282 |  | 0.92 (0.86-0.98) | 0.015 |  |  | 1.13 (0.96-1.34) | 0.154 |  | 1.01 (0.85-1.21) | 0.886 |  |
| Southwest | 0.98 (0.83-1.17) | 0.830 |  | 0.97 (0.82-1.16) | 0.754 |  |  | 1.19 (0.79-1.82) | 0.406 |  | 1.10 (0.72-1.68) | 0.649 |  |
| **Adjusted median family income** |  |  |  |  |  |  |  |  |  |  |  |  |  |
| $(~74400] | 1 reference |  |  | 1 reference |  |  |  | 1 reference |  |  | 1 reference |  |  |
| $(74400~) | 0.80 (0.75-0.85) | <0.001 |  | 0.83 (0.78-0.89) | <0.001 |  |  | 0.78 (0.67-0.92) | 0.002 |  | 0.81 (0.68-0.96) | 0.013 |  |
| **Insurance** |  |  |  |  |  |  |  |  |  |  |  |  |  |
| Any Medicaid | 1 reference |  |  | 1 reference |  |  |  | 1 reference |  |  | 1 reference |  |  |
| Insured | 0.71 (0.63-0.80) | <0.001 |  | 0.68 (0.60-0.77) | <0.001 |  |  | 0.78 (0.58-1.06) | 0.109 |  | 0.74 (0.54-1.01) | 0.056 |  |
| Uninsured | 0.81 (0.71-0.92) | 0.001 |  | 0.79 (0.68-0.91) | 0.002 |  |  | 0.81 (0.58-1.13) | 0.223 |  | 0.69 (0.48-1.01) | 0.057 |  |
| **Population density** |  |  |  |  |  |  |  |  |  |  |  |  |  |
| Counties | 1 reference |  |  | 1 reference |  |  |  | 1 reference |  |  | 1 reference |  |  |
| Urban/Rural | 1.11 (1.02-1.21) | 0.013 |  | 1.07 (0.97-1.17) | 0.167 |  |  | 1.06 (0.85-1.32) | 0.630 |  | 0.99 (0.78-1.27) | 0.963 |  |
| **Prior cancer diagnosis** |  |  |  |  |  |  |  |  |  |  |  |  |  |
| No | 1 reference |  |  | 1 reference |  |  |  | 1 reference |  |  | 1 reference |  |  |
| 1 only | 2.00 (1.87-2.15) | <0.001 |  | 1.55 (1.44-1.66) | <0.001 |  |  | 2.51 (2.11-2.97) | <0.001 |  | 2.04 (1.72-2.43) | <0.001 |  |
| 2 or more | 3.45 (3.07-3.87) | <0.001 |  | 2.36 (2.10-2.66) | <0.001 |  |  | 2.92 (2.11-4.05) | <0.001 |  | 2.12 (1.52-2.96) | <0.001 |  |
| **Marital status** |  |  |  |  |  |  |  |  |  |  |  |  |  |
| Married | 1 reference |  |  | 1 reference |  |  |  | 1 reference |  |  | 1 reference |  |  |
| Never married | 1.08 (0.98-1.18) | 0.107 |  | 1.40 (1.28-1.55) | <0.001 |  |  | 0.80 (0.62-1.03) | 0.089 |  | 1.00 (0.77-1.30) | 0.973 |  |
| Separated/Widowed/Divorced | 1.76 (1.63-1.89) | <0.001 |  | 1.48 (1.37-1.60) | <0.001 |  |  | 1.38 (1.14-1.68) | 0.001 |  | 1.24 (1.01-1.53) | 0.037 |  |
| **Age at diagnosis, years** |  |  |  |  |  |  |  |  |  |  |  |  |  |
| ≦59 | 1 reference |  |  | 1 reference |  |  |  | 1 reference |  |  | 1 reference |  |  |
| 60-74 | 2.51 (2.32-2.72) | <0.001 |  | 2.25 (2.07-2.43) | <0.001 |  |  | 2.10 (1.74-2.54) | <0.001 |  | 1.65 (1.35-2.00) | <0.001 |  |
| 75-84 | 5.88 (5.41-6.44) | <0.001 |  | 4.51 (4.11-4.95) | <0.001 |  |  | 4.11 (3.28-5.14) | <0.001 |  | 2.57 (2.02-3.27) | <0.001 |  |
| 85+ | 10.95 (9.31-12.88) | <0.001 |  | 6.87 (5.78-8.16) | <0.001 |  |  | 8.18 (5.37-1.46) | <0.001 |  | 3.50 (2.24-5.47) | <0.001 |  |
| **Race** |  |  |  |  |  |  |  |  |  |  |  |  |  |
| White | 1 reference |  |  | 1 reference |  |  |  | 1 reference |  |  | 1 reference |  |  |
| Black | 1.08 (0.98-1.18) | 0.133 |  | 1.04 (0.94-1.14) | 0.500 |  |  | 0.85 (0.65-1.11) | 0.239 |  | 0.82 (0.62-1.09) | 0.165 |  |
| Other | 0.70 (0.61-0.82) | <0.001 |  | 0.81 (0.70-0.95) | 0.008 |  |  | 0.79 (0.55-1.12) | 0.186 |  | 0.85 (0.59-1.22) | 0.367 |  |
| **Sex** |  |  |  |  |  |  |  |  |  |  |  |  |  |
| Female | 1 reference |  |  | 1 reference |  |  |  | 1 reference |  |  | 1 reference |  |  |
| Male | 1.27 (1.19-1.35) | <0.001 |  | 1.31 (1.23-1.41) | <0.001 |  |  | 1.46 (1.23-1.74) | <0.001 |  | 1.34 (1.12-1.61) | 0.001 |  |
| **Grade** |  |  |  |  |  |  |  |  |  |  |  |  |  |
| I+II | 1 reference |  |  | 1 reference |  |  |  | 1 reference |  |  | 1 reference |  |  |
| III+IV | 1.24 (1.14-1.34) | <0.001 |  | 1.16 (1.08-1.26) | <0.001 |  |  | 2.38 (2.00-2.83) | <0.001 |  | 2.22 (1.85-2.66) | <0.001 |  |
| **Laterality** |  |  |  |  |  |  |  |  |  |  |  |  |  |
| Left | 1 reference |  |  | 1 reference |  |  |  | 1 reference |  |  | 1 reference |  |  |
| Right | 1.00 (0.94-1.06) | 0.948 |  | 1.00 (0.95-1.07) | 0.895 |  |  | 1.19 (1.02-1.40) | 0.029 |  | 1.22 (1.04-1.43) | 0.013 |  |
| **Histological type** |  |  |  |  |  |  |  |  |  |  |  |  |  |
| ccRCC | 1 reference |  |  | 1 reference |  |  |  | 1 reference |  |  | 1 reference |  |  |
| paRCC | 0.93 (0.86-1.12) | 0.285 |  | 0.91 (0.83-0.99) | 0.028 |  |  | 0.90 (0.72-1.12) | 0.349 |  | 0.75 (0.60-0.94) | 0.012 |  |
| chRCC | 0.68 (0.59-0.79) | <0.001 |  | 0.66 (0.56-0.77) | <0.001 |  |  | 0.33 (0.19-0.56) | <0.001 |  | 0.31 (0.18-0.53) | <0.001 |  |
| Other RCC | 1.17 (1.08-1.26) | <0.001 |  | 1.02 (0.94-1.10) | 0.716 |  |  | 1.02 (0.84-1.24) | 0.842 |  | 0.89 (0.73-1.09) | 0.274 |  |
| **Tumor size, cm** |  |  |  |  |  |  |  |  |  |  |  |  |  |
| ≦2cm | 1 reference |  |  | 1 reference |  |  |  | 1 reference |  |  | 1 reference |  |  |
| 2-3cm | 1.33 (1.22-1.44) | <0.001 |  | 1.18 (1.08-1.28) | <0.001 |  |  | 1.46 (1.15-1.85) | 0.002 |  | 1.27 (1.00-1.61) | 0.053 |  |
| 3-4cm | 1.75 (1.60-1.91) | <0.001 |  | 1.49 (1.37-1.63) | <0.001 |  |  | 2.07 (1.61-2.66) | <0.001 |  | 1.74 (1.35-2.24) | <0.001 |  |
| 4-7cm | 2.07 (1.89-2.27) | <0.001 |  | 1.93 (1.75-2.12) | <0.001 |  |  | 4.12 (3.26-5.19) | <0.001 |  | 3.75 (2.95-4.75) | <0.001 |  |
| **Surgery type** |  |  |  |  |  |  |  |  |  |  |  |  |  |
| Partial nephrectomy | 1 reference |  |  | 1 reference |  |  |  | 1 reference |  |  | 1 reference |  |  |
| Local ablation | 2.51 (2.34-2.70) | <0.001 |  | 1.66 (1.53-1.81) | <0.001 |  |  | 2.65 (2.21-3.18) | <0.001 |  | 2.41 (1.95-2.97) | <0.001 |  |
| RCC, Renal cell carcinoma; HR, Hazard ratios; CI, confidence intervals.  ǂUnivariable and multivariate Cox regression analysis.  ¶ Univariable and multivariate cause-specific Cox regression analysis. | | | | | | | | | | | | | |

| **Supplementary Table 4.** Prognostic factors for overall and cancer-specific mortality of T1N0M0 renal cell carcinoma patients with treatment of partial nephrectomy and local ablation (Inverse probability of imputed treatment weighted population) | | | | | | | | | | | | |
| --- | --- | --- | --- | --- | --- | --- | --- | --- | --- | --- | --- | --- |
| **Covariables** | **Overall mortality ǂ** | | | | |  | **RCC-specific mortality ¶** | | | | | |
|  | **Unadjusted**  **HR (95%CI)** | ***P-value*** |  | **Adjusted**  **HR (95%CI)** | ***P-value*** |  | **Unadjusted**  **HR (95%CI)** | ***P-value*** |  | **Adjusted**  **HR (95%CI)** | ***P-value*** |  |
| **Year at diagnosis** |  |  |  |  |  |  |  |  |  |  |  |  |
| 2004-2007 | 1 reference |  |  | 1 reference |  |  | 1 reference |  |  | 1 reference |  |  |
| 2008-2012 | 0.84 (0.74-0.95) | 0.005 |  | 0.91 (0.77-1.08) | 0.300 |  | 0.93 (0.67-1.30) | 0.670 |  | 0.89 (0.57-1.39) | 0.617 |  |
| 2013-2016 | 0.82 (0.67-0.99) | 0.043 |  | 0.88 (0.70-1.10) | 0.256 |  | 0.84 (0.51-1.39) | 0.500 |  | 0.78 (0.44-1.40) | 0.406 |  |
| **Region** |  |  |  |  |  |  |  |  |  |  |  |  |
| East | 1 reference |  |  | 1 reference |  |  | 1 reference |  |  | 1 reference |  |  |
| Northern Plains | 1.09 (0.94-1.28) | 0.265 |  | 1.11 (0.94-1.30) | 0.225 |  | 1.21 (0.80-1.83) | 0.361 |  | 1.17 (0.77-1.80) | 0.462 |  |
| Pacific Coast | 0.99 (0.88-1.12) | 0.862 |  | 0.94 (0.83-1.07) | 0.340 |  | 1.13 (0.82-1.55) | 0.455 |  | 1.05 (0.75-1.48) | 0.766 |  |
| Southwest | 0.93 (0.70-1.23) | 0.599 |  | 1.01 (0.76-1.35) | 0.925 |  | 1.17 (0.58-2.35) | 0.665 |  | 1.16 (0.56-2.39) | 0.694 |  |
| **Adjusted median family income** |  |  |  |  |  |  |  |  |  |  |  |  |
| $(~74400] | 1 reference |  |  | 1 reference |  |  | 1 reference |  |  | 1 reference |  |  |
| $(74400~) | 0.80 (0.72-0.89) | <0.001 |  | 0.79 (0.70-0.88) | <0.001 |  | 0.65 (0.49-0.85) | 0.002 |  | 0.61 (0.45-0.83) | 0.002 |  |
| **Insurance** |  |  |  |  |  |  |  |  |  |  |  |  |
| Any Medicaid | 1 reference |  |  | 1 reference |  |  | 1 reference |  |  | 1 reference |  |  |
| Insured | 0.80 (0.66-0.98) | 0.028 |  | 0.74 (0.61-0.91) | 0.003 |  | 1.11 (0.64-1.94) | 0.713 |  | 1.05 (0.59-1.85) | 0.879 |  |
| Uninsured | 0.96 (0.77-1.19) | 0.698 |  | 0.82 (0.64-1.06) | 0.130 |  | 1.09 (0.58-2.03) | 0.791 |  | 0.96 (0.47-1.95) | 0.899 |  |
| **Population density** |  |  |  |  |  |  |  |  |  |  |  |  |
| Counties | 1 reference |  |  | 1 reference |  |  | 1 reference |  |  | 1 reference |  |  |
| Urban/Rural | 1.03 (0.89-1.20) | 0.694 |  | 0.97 (0.82-1.15) | 0.755 |  | 1.14 (0.77-1.68) | 0.511 |  | 0.99 (0.64-1.51) | 0.951 |  |
| **Prior cancer diagnosis** |  |  |  |  |  |  |  |  |  |  |  |  |
| No | 1 reference |  |  | 1 reference |  |  | 1 reference |  |  | 1 reference |  |  |
| 1 only | 1.60 (1.42-1.80) | <0.001 |  | 1.49 (1.32-1.68) | <0.001 |  | 2.12 (1.58-2.86) | <0.001 |  | 2.08 (1.54-2.82) | <0.001 |  |
| 2 or more | 2.50 (2.09-2.99) | <0.001 |  | 2.26 (1.88-2.71) | <0.001 |  | 2.44 (1.49-4.00) | <0.001 |  | 2.27 (1.37-3.76) | 0.001 |  |
| **Marital status** |  |  |  |  |  |  |  |  |  |  |  |  |
| Married | 1 reference |  |  | 1 reference |  |  | 1 reference |  |  | 1 reference |  |  |
| Never married | 1.07 (0.90-1.27) | 0.471 |  | 1.37 (1.15-1.65) | 0.001 |  | 0.73 (0.44-1.21) | 0.223 |  | 0.96 (0.57-1.62) | 0.865 |  |
| Separated/Widowed/Divorced | 1.54 (1.37-1.74) | <0.001 |  | 1.41 (1.24-1.61) | <0.001 |  | 1.21 (0.87-1.69) | 0.268 |  | 1.15 (0.80-1.64) | 0.457 |  |
| **Age at diagnosis, years** |  |  |  |  |  |  |  |  |  |  |  |  |
| ≦59 | 1 reference |  |  | 1 reference |  |  | 1 reference |  |  | 1 reference |  |  |
| 60-74 | 1.92 (1.62-2.28) | <0.001 |  | 1.82 (1.53-2.17) | <0.001 |  | 1.97 (1.26-3.07) | <0.001 |  | 1.69 (1.07-2.65) | 0.024 |  |
| 75-84 | 3.80 (3.20-4.52) | <0.001 |  | 3.42 (2.85-4.09) | <0.001 |  | 3.44 (2.18-5.45) | <0.001 |  | 2.73 (1.70-4.39) | <0.001 |  |
| 85+ | 6.53 (5.10-8.37) | <0.001 |  | 5.45 (4.21-7.07) | <0.001 |  | 5.91 (3.05-11.43) | <0.001 |  | 3.97 (1.99-7.91) | <0.001 |  |
| **Race** |  |  |  |  |  |  |  |  |  |  |  |  |
| White | 1 reference |  |  | 1 reference |  |  | 1 reference |  |  | 1 reference |  |  |
| Black | 0.93 (0.78-1.10) | 0.378 |  | 0.96 (0.80-1.15) | 0.645 |  | 0.84 (0.53-1.35) | 0.476 |  | 0.86 (0.52-1.42) | 0.559 |  |
| Other | 0.85 (0.65-1.10) | 0.216 |  | 0.90 (0.69-1.18) | 0.460 |  | 1.33 (0.76-2.33) | 0.313 |  | 1.39 (0.78-2.48) | 0.271 |  |
| **Sex** |  |  |  |  |  |  |  |  |  |  |  |  |
| Female | 1 reference |  |  | 1 reference |  |  | 1 reference |  |  | 1 reference |  |  |
| Male | 1.14 (1.02-1.27) | 0.024 |  | 1.23 (1.09-1.39) | 0.001 |  | 1.19 (0.88-1.60) | 0.262 |  | 1.13 (0.82-1.56) | 0.446 |  |
| **Grade** |  |  |  |  |  |  |  |  |  |  |  |  |
| I+II | 1 reference |  |  | 1 reference |  |  | 1 reference |  |  | 1 reference |  |  |
| III+IV | 1.21 (0.99-1.49) | 0.064 |  | 1.16 (0.94-1.42) | 0.162 |  | 1.79 (1.13-2.82) | 0.013 |  | 1.66 (1.04-2.64) | 0.032 |  |
| **Laterality** |  |  |  |  |  |  |  |  |  |  |  |  |
| Left | 1 reference |  |  | 1 reference |  |  | 1 reference |  |  | 1 reference |  |  |
| Right | 0.98 (0.89-1.09) | 0.771 |  | 0.99 (0.89-1.10) | 0.791 |  | 1.35 (1.02-1.79) | 0.038 |  | 1.38 (1.04-1.84) | 0.026 |  |
| **Histological type** |  |  |  |  |  |  |  |  |  |  |  |  |
| ccRCC | 1 reference |  |  | 1 reference |  |  | 1 reference |  |  | 1 reference |  |  |
| paRCC | 0.85 (0.73-0.99) | 0.041 |  | 0.84 (0.72-0.99) | 0.037 |  | 0.77 (0.51-1.16) | 0.202 |  | 0.83 (0.54-1.26) | 0.375 |  |
| chRCC | 0.90 (0.68-1.19) | 0.454 |  | 0.75 (0.56-1.00) | 0.052 |  | 0.35 (0.11-1.05) | 0.062 |  | 0.30 (0.10-0.94) | 0.038 |  |
| Other RCC | 0.96 (0.85-1.09) | 0.517 |  | 0.96 (0.84-1.10) | 0.556 |  | 0.84 (0.60-1.18) | 0.309 |  | 0.86 (0.60-1.22) | 0.388 |  |
| **Tumor size, cm** |  |  |  |  |  |  |  |  |  |  |  |  |
| ≦2cm | 1 reference |  |  | 1 reference |  |  | 1 reference |  |  | 1 reference |  |  |
| 2-3cm | 1.36 (1.18-1.57) | <0.001 |  | 1.24 (1.08-1.44) | 0.003 |  | 1.41 (0.94-2.10) | 0.094 |  | 1.29 (0.86-1.93) | 0.224 |  |
| 3-4cm | 1.96 (1.68-2.28) | <0.001 |  | 1.68 (1.44-1.96) | <0.001 |  | 2.23 (1.47-3.38) | <0.001 |  | 1.90 (1.24-2.90) | 0.003 |  |
| 4-7cm | 2.70 (2.24-3.25) | <0.001 |  | 2.08 (1.72-2.52) | <0.001 |  | 4.38 (2.78-6.92) | <0.001 |  | 3.45 (2.15-5.53) | <0.001 |  |
| **Surgery type** |  |  |  |  |  |  |  |  |  |  |  |  |
| Partial nephrectomy | 1 reference |  |  | 1 reference |  |  | 1 reference |  |  | 1 reference |  |  |
| Local ablation | 1.56 (1.40-1.73) | <0.001 |  | 1.56 (1.40-1.74) | <0.001 |  | 2.17 (1.61-2.91) | <0.001 |  | 2.21 (1.63-2.98) | <0.001 |  |
| RCC, Renal cell carcinoma; HR, Hazard ratios; CI, confidence intervals.  ǂUnivariable and multivariate Cox regression analysis.  ¶ Univariable and multivariate cause-specific Cox regression analysis. | | | | | | | | | | | | |

| **Supplementary Table 5.** Subgroup analysis for the impact of different treatments of partial nephrectomy (as a reference [1]) vs. local ablation in subgroup population on overall and cancer-specific mortality of patients with T1N0M0 renal cell carcinoma (unweighted population). | | | | | | | | | |
| --- | --- | --- | --- | --- | --- | --- | --- | --- | --- |
| **Subgroup population** | **Overall mortality ǂ** | | | |  | **RCC-specific mortality ¶** | | | |
|  | **Unadjusted**  **HR (95%CI)** | ***P***  ***-value*** | **Adjusted**  **HR (95%CI)** | ***P***  ***-value*** |  | **Unadjusted**  **HR (95%CI)** | ***P***  ***-value*** | **Adjusted**  **HR (95%CI)** | ***P***  ***-value*** |
| **Year at diagnosis** |  |  |  |  |  |  |  |  |  |
| 2004-2007 | 2.29 (2.03-2.58) | <0.001 | 1.48 (1.30-1.69) | <0.001 |  | 2.32 (1.69-3.18) | <0.001 | 1.98 (1.39-2.82) | <0.001 |
| 2008-2012 | 2.55 (2.30-2.82) | <0.001 | 1.74 (1.54-1.96) | <0.001 |  | 2.91 (2.26-3.74) | <0.001 | 2.80 (2.08-3.77) | <0.001 |
| 2013-2016 | 3.31 (2.71-4.03) | <0.001 | 2.66 (2.09-3.38) | <0.001 |  | 2.78 (1.73-4.48) | <0.001 | 2.92 (1.59-5.36) | 0.001 |
| **Region** |  |  |  |  |  |  |  |  |  |
| East | 2.39 (2.12-2.68) | <0.001 | 1.59 (1.40-1.82) | <0.001 |  | 2.48 (1.82-3.38) | <0.001 | 2.16 (1.52-3.06) | <0.001 |
| Northern Plains | 2.63 (2.18-3.16) | <0.001 | 1.84 (1.49-2.28) | <0.001 |  | 2.94 (1.81-4.78) | <0.001 | 2.73 (1.55-4.80) | <0.001 |
| Pacific Coast | 2.59 (2.32-2.89) | <0.001 | 1.76 (1.55-2.00) | <0.001 |  | 2.59 (1.98-3.39) | <0.001 | 2.44 (1.77-3.36) | <0.001 |
| Southwest | 2.36 (1.62-3.46) | <0.001 | 1.74 (1.12-2.69) | 0.013 |  | 3.87 (1.69-8.86) | 0.001 | 4.28 (1.45-12.6) | 0.008 |
| **Adjusted median family income** |  |  |  |  |  |  |  |  |  |
| $(~74400] | 2.50 (2.26-2.76) | <0.001 | 1.69 (1.51-1.89) | <0.001 |  | 2.94 (2.30-3.76) | <0.001 | 2.73 (2.05-3.63) | <0.001 |
| $(74400~) | 2.52 (2.28-2.80) | <0.001 | 1.69 (1.50-1.90) | <0.001 |  | 2.36 (1.80-3.09) | <0.001 | 2.12 (1.56-2.90) | <0.001 |
| **Insurance** |  |  |  |  |  |  |  |  |  |
| Any Medicaid | 2.48 (1.94-3.17) | <0.001 | 2.38 (1.77-3.20) | <0.001 |  | 1.82 (0.92-3.57) | 0.083 | 1.94 (0.80-4.68) | 0.140 |
| Insured | 2.66 (2.43-2.91) | <0.001 | 1.77 (1.60-1.96) | <0.001 |  | 2.83 (2.28-3.52) | <0.001 | 2.61 (2.03-3.37) | <0.001 |
| Uninsured | 2.22 (1.92-2.55) | <0.001 | 1.44 (1.23-1.68) | <0.001 |  | 2.49 (1.70-3.64) | <0.001 | 2.14 (1.39-3.28) | 0.001 |
| **Population density** |  |  |  |  |  |  |  |  |  |
| Counties | 2.57 (2.37-2.77) | <0.001 | 1.70 (1.55-1.86) | <0.001 |  | 2.59 (2.13-3.16) | <0.001 | 2.30 (1.82-2.89) | <0.001 |
| Urban/Rural | 2.23 (1.83-2.70) | <0.001 | 1.71 (1.38-2.13) | <0.001 |  | 3.05 (1.91-4.86) | <0.001 | 3.27 (1.91-5.62) | <0.001 |
| **Prior cancer diagnosis** |  |  |  |  |  |  |  |  |  |
| No | 2.55 (2.32-2.79) | <0.001 | 1.71 (1.54-1.90) | <0.001 |  | 2.47 (1.93-3.16) | <0.001 | 2.31 (1.74-3.07) | <0.001 |
| 1 only | 1.92 (1.68-2.19) | <0.001 | 1.67 (1.44-1.94) | <0.001 |  | 2.15 (1.58-2.92) | <0.001 | 2.46 (1.73-3.51) | <0.001 |
| 2 or more | 1.77 (1.40-2.23) | <0.001 | 1.63 (1.25-2.14) | <0.001 |  | 2.52 (1.34-4.71) | 0.004 | 2.70 (1.29-5.63) | 0.008 |
| **Marital status** |  |  |  |  |  |  |  |  |  |
| Married | 2.50 (2.27-2.75) | <0.001 | 1.67 (1.50-1.87) | <0.001 |  | 2.72 (2.17-3.42) | <0.001 | 2.53 (1.94-3.30) | <0.001 |
| Never married | 2.51 (2.03-3.10) | <0.001 | 1.86 (1.46-2.37) | <0.001 |  | 2.62 (1.45-4.71) | 0.001 | 2.51 (1.25-5.04) | 0.010 |
| Separated/Widowed/Divorced | 2.15 (1.87-2.47) | <0.001 | 1.62 (1.38-1.89) | <0.001 |  | 1.87 (1.26-2.77) | 0.002 | 1.78 (1.14-2.78) | 0.011 |
| **Age at diagnosis, years** |  |  |  |  |  |  |  |  |  |
| ≦59 | 2.40 (1.98-2.92) | <0.001 | 2.18 (1.77-2.68) | <0.001 |  | 2.05 (1.26-3.34) | 0.004 | 2.38 (1.40-4.04) | 0.001 |
| 60-74 | 1.77 (1.58-1.98) | <0.001 | 1.74 (1.54-1.96) | <0.001 |  | 1.94 (1.47-2.57) | <0.001 | 2.23 (1.64-3.04) | <0.001 |
| 75-84 | 1.41 (1.242-1.60) | <0.001 | 1.53 (1.33-1.77) | <0.001 |  | 1.86 (1.33-2.59) | <0.001 | 2.30 (1.57-3.36) | <0.001 |
| 85+ | 1.10 (0.81-1.48) | 0.551 | 1.10 (0.76-1.60) | 0.624 |  | 1.39 (0.62-3.09) | 0.424 | 4.73 (1.29-17.4) | 0.019 |
| **Race** |  |  |  |  |  |  |  |  |  |
| White | 2.52 (2.33-2.72) | <0.001 | 1.69 (1.55-1.85) | <0.001 |  | 2.45 (2.00-2.99) | <0.001 | 2.21 (1.76-2.79) | <0.001 |
| Black | 2.10 (1.69-2.61) | <0.001 | 1.62 (1.27-2.07) | <0.001 |  | 2.99 (1.70-5.24) | <0.001 | 3.54 (1.81-6.94) | <0.001 |
| Other | 3.56 (2.55-4.96) | <0.001 | 2.25 (1.53-3.32) | <0.001 |  | 6.77 (3.34-13.71) | <0.001 | 8.14 (3.00-22.05) | <0.001 |
| **Sex** |  |  |  |  |  |  |  |  |  |
| Female | 2.78 (2.45-3.16) | <0.001 | 1.91 (1.66-2.20) | <0.001 |  | 2.96 (2.13-4.13) | <0.001 | 2.62 (1.80-3.80) | <0.001 |
| Male | 2.38 (2.18-2.60) | <0.001 | 1.61 (1.46-1.79) | <0.001 |  | 2.51 (2.02-3.12) | <0.001 | 2.36 (1.83-3.05) | <0.001 |
| **Grade** |  |  |  |  |  |  |  |  |  |
| I+II | 2.62 (2.38-2.88) | <0.001 | 1.69 (1.53-1.87) | <0.001 |  | 3.72 (2.91-4.75) | <0.001 | 2.63 (2.02-3.42) | <0.001 |
| III+IV | 2.19 (1.69-2.84) | <0.001 | 1.72 (1.31-2.26) | <0.001 |  | 2.01 (1.17-3.46) | 0.012 | 2.00 (1.14-3.52) | 0.016 |
| **Laterality** |  |  |  |  |  |  |  |  |  |
| Left | 2.52 (2.27-2.79) | <0.001 | 1.62 (1.44-1.82) | <0.001 |  | 2.53 (1.92-3.33) | <0.001 | 2.30 (1.67-3.16) | <0.001 |
| Right | 2.50 (2.26-2.77) | <0.001 | 1.79 (1.59-2.00) | <0.001 |  | 2.76 (2.17-3.52) | <0.001 | 2.67 (2.01-3.53) | <0.001 |
| **Histological type** |  |  |  |  |  |  |  |  |  |
| ccRCC | 2.76 (2.51-3.05) | <0.001 | 1.90 (1.70-2.12) | <0.001 |  | 2.79 (2.20-3.54) | <0.001 | 2.50 (1.91-3.28) | <0.001 |
| paRCC | 2.05 (1.71-2.47) | <0.001 | 1.65 (1.34-2.03) | <0.001 |  | 2.52 (1.58-4.01) | <0.001 | 2.85 (1.67-4.88) | <0.001 |
| chRCC | 3.55 (2.44-5.17) | <0.001 | 1.75 (1.13-2.71) | 0.012 |  | 2.38 (0.53-10.65) | 0.258 | 0.54 (0.08-3.74) | 0.535 |
| Other RCC | 2.13 (1.85-2.45) | <0.001 | 1.38 (1.16-1.63) | <0.001 |  | 2.32 (1.62-3.33) | <0.001 | 2.19 (1.40-3.42) | 0.001 |
| **Tumor size, cm** |  |  |  |  |  |  |  |  |  |
| ≦2cm | 2.03 (1.72-2.39) | <0.001 | 1.28 (1.07-1.54) | 0.007 |  | 3.04 (1.97-4.67) | <0.001 | 2.02 (1.24-3.27) | 0.004 |
| 2-3cm | 2.45 (2.17-2.76) | <0.001 | 1.53 (1.34-1.76) | <0.001 |  | 2.87 (2.08-3.97) | <0.001 | 2.04 (1.39-2.99) | <0.001 |
| 3-4cm | 3.32 (2.90-3.79) | <0.001 | 2.30 (1.96-2.70) | <0.001 |  | 3.68 (2.59-5.22) | <0.001 | 3.91 (2.58-5.92) | <0.001 |
| 4-7cm | 3.14 (2.58-3.80) | <0.001 | 2.09 (1.69-2.58) | <0.001 |  | 2.85 (1.90-4.27) | <0.001 | 2.41 (1.54-3.77) | <0.001 |
| RCC, Renal cell carcinoma; HR, Hazard ratios; CI, confidence intervals.  ǂUnivariable (unadjusted model) and multivariate (full covariables adjusted model) Cox regression analysis in each subgroup cohort.  ¶ Univariable (unadjusted model) and multivariate (full covariables adjusted model) cause-specific Cox regression analysis in each subgroup cohort. | | | | | | | | | |
